# A review of botany, phytochemistry, pharmacology, and applications of the herb with the homology of medicine and food: *Ligustrum lucidum* W.T. Aiton

**DOI:** 10.3389/fphar.2024.1330732

**Published:** 2024-06-12

**Authors:** Liping Chen, Dong Huang, Lin Jiang, Jihong Yang, Xiaoyu Shi, Rong Wang, Wenbin Li

**Affiliations:** ^1^ Department of Pharmacy, The 940th Hospital of Joint Logistic Support Force of PLA, Lanzhou, China; ^2^ School of Medicine, Tibet University, Lhasa, China; ^3^ School of Pharmacy, Gansu University of Chinese Medicine, Lanzhou, Gansu, China

**Keywords:** *Ligustrum lucidum* W.T. aiton, homology of medicine and food, nutritional value and chemical composition, pharmacological activity, applications

## Abstract

*Ligustrum lucidum* W.T. Aiton is an outstanding herb with the homology of medicine and food. Its ripe fruits are traditionally used as an important tonic for kidneys and liver in China. *Ligustrum lucidum* W.T. Aiton is rich in nutritional components and a variety of bioactive ingredients. A total of 206 compounds have been isolated and identified, they mainly include flavonoids, phenylpropanoids, iridoid glycosides, and triterpenoids. These compounds exert anti-osteoporosis, anti-tumor, liver protective, antioxidant, anti-inflammatory, and immunomodulatory effects. *Ligustrum lucidum* W.T. Aiton has been traditionally used to treat many complex diseases, including osteoporotic bone pain, rheumatic bone, cancer, related aging symptoms, and so on. In the 2020 Edition of Chinese Pharmacopoeia, there are more than 100 prescriptions containing *L. lucidum* W.T. Aiton. Among them, some classical preparations including Er Zhi Wan and Zhenqi fuzheng formula, are used in the treatment of various cancers with good therapeutic effects. Additionally, *L. lucidum* W.T. Aiton has also many excellent applications for functional food, ornamental plants, bioindicator of air pollution, algicidal agents, and feed additives. *Ligustrum lucidum* W.T. Aiton has rich plant resources. However, the application potential of it has not been fully exploited. We hope that this paper provides a theoretical basis for the high-value and high-connotation development of *L. lucidum* W.T. Aiton in the future.

## 1 Introduction

*Ligustrum lucidum* W.T. Aiton (*L. lucidum*), also known as glossy privet, is a popular species of flowering plant that belongs to the olive family Oleaceae. It has been cultivated and distributed in most areas of China and many parts of the world since ancient times, and has rich plant resources (Liu et al., 2019). With the prosperous development of the social economy and the persistent improvement of people’s living standards, it has become increasingly popular globally that preventing ailments via diet is an effective avenue to ensure the health of people in recent decades (Duque-Buitrago et al., 2023). Medicine food homology (MFH) materials just cater to the demand. The theory of MFH is long-standing in China, which systematically elaborates on how to combine foods and drugs for health and medical properties (Gong et al., 2020). It means that traditional Chinese medicine (TCM) and food occur simultaneously. MFH combines the virtue of drug and food perfectly and thus can be utilized both for drug and food. Besides the effect on the prevention and treatment of ailments, MFH materials also usually possess many other nutritional values and healthcare functions (Lerner and Benzvi, 2021). To ensure the safe application, the China Food and Drug Administration (CFDA) has authorized specific provisions on MFH items. *Ligustrum lucidum* is not only a unique herbal remedy for tonifying the liver and kidney in TCM, but also a significant MFH material released by CFDA. In other words, *L. lucidum* has been applied as both food and medicine in numerous conventional medical systems throughout history.

As a good MFH material, it is broadly recognized that *L. lucidum* possesses the function of health preservation and prolonging the life span through invigorating the liver and kidney deficiency, and is also widely used in healthy food. Additionally, *L. lucidum* is one of the most popular green dietary supplements utilized by tumor patients all over the world. It is also consumed as herbal tea in Korea and India. It is noteworthy that *L. lucidum* also possesses many unique properties, such as being a health promoter utilized by animals, a general bioindicator of air pollution environments, the treatment of eutrophic water, and an outstanding ornamental plant, and is widely used in food, livestock husbandry, gardens, and other fields.

**FIGURE 1:**
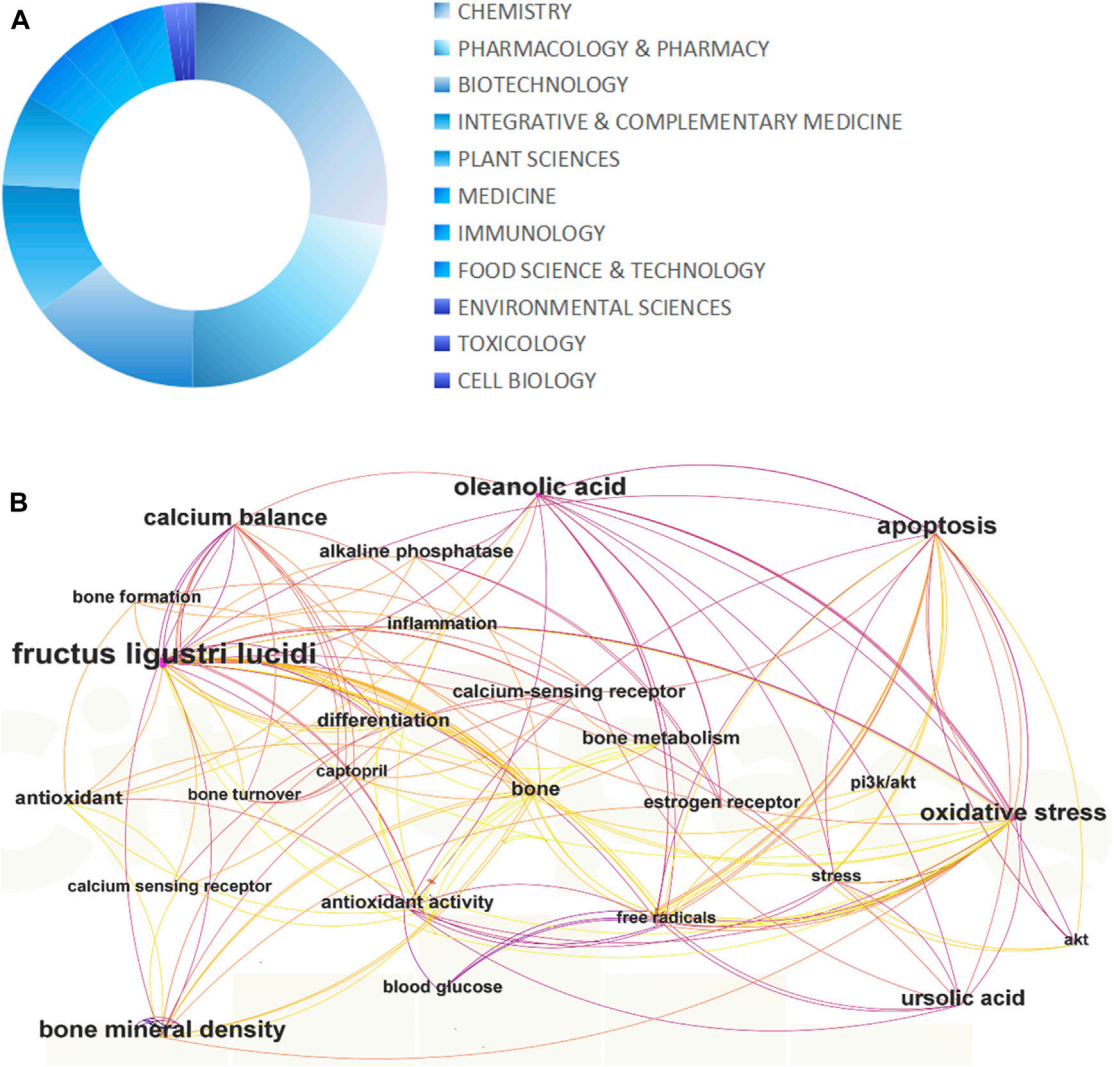
Number of articles (>5 articles) about different research interests of *Ligustrum lucidum* W.T. Aiton as of April 2024, based on the Web of Science **(A)**, analysis of the co-occurrence links between keywords that occur at least five times in the selected articles **(B)**.

In conclusion, as a kind of potential treasure trove for medicine, functional foods, feed additives, and other industries, *L. lucidum* has extremely high exploitation value. In the present study, we used the VOS-viewer to analyze the co-occurrence of links between keywords of the publications related to *L. lucidum* in the Web of Science, which is a total of 219 pieces of relevant literature up to April 2024 (Figure 1). This review not only focuses on the phytochemicals and pharmacological properties of *L. lucidum*, but also for the first time reviews the application of *L. lucidum* in terms of economics based on its translational applications. We hope that this paper provides a theoretical basis for high-value and high-connotation development of *L. lucidum* in the future.

## 2 Botanical description

*Ligustrum lucidum* W.T. Aiton (also named “Nvzhenzi” in China and “Dang-gwang-na-mu” in Korea), belongs to the Oleaceae family (Jang et al., 2020). It was recorded in the Compendium of Materia Medica that *L. lucidum* kept verdant rather than withering even in the depth of winter, seemed to have the virtue of a chaste woman. And thus, the name of Nv Zhen was obtained since ancient times in China. According to “The Plant List,” L. lu*cidum* is the only accepted name for this MFH plant, with other 18 synonyms. The synonyms with the highest confidence levels include *Esquirolia sinensis* H. Lév., *Ligustrum esquirolii* H. Lév., *Ligustrum magnoliifolium* Dippel, *Ligustrum roxburghii* Blume, *Ligustrum wallichii* Vis., *Olea chinensis* Sweet, *Olea clavata* G. Don, *Phillyrea paniculata* Roxb., *Phillyrea terminalis* B. Heyne ex Wall., and *Visiania paniculata* (Roxb.) DC. (https://mpns.science.kew.org/).

**FIGURE 2:**
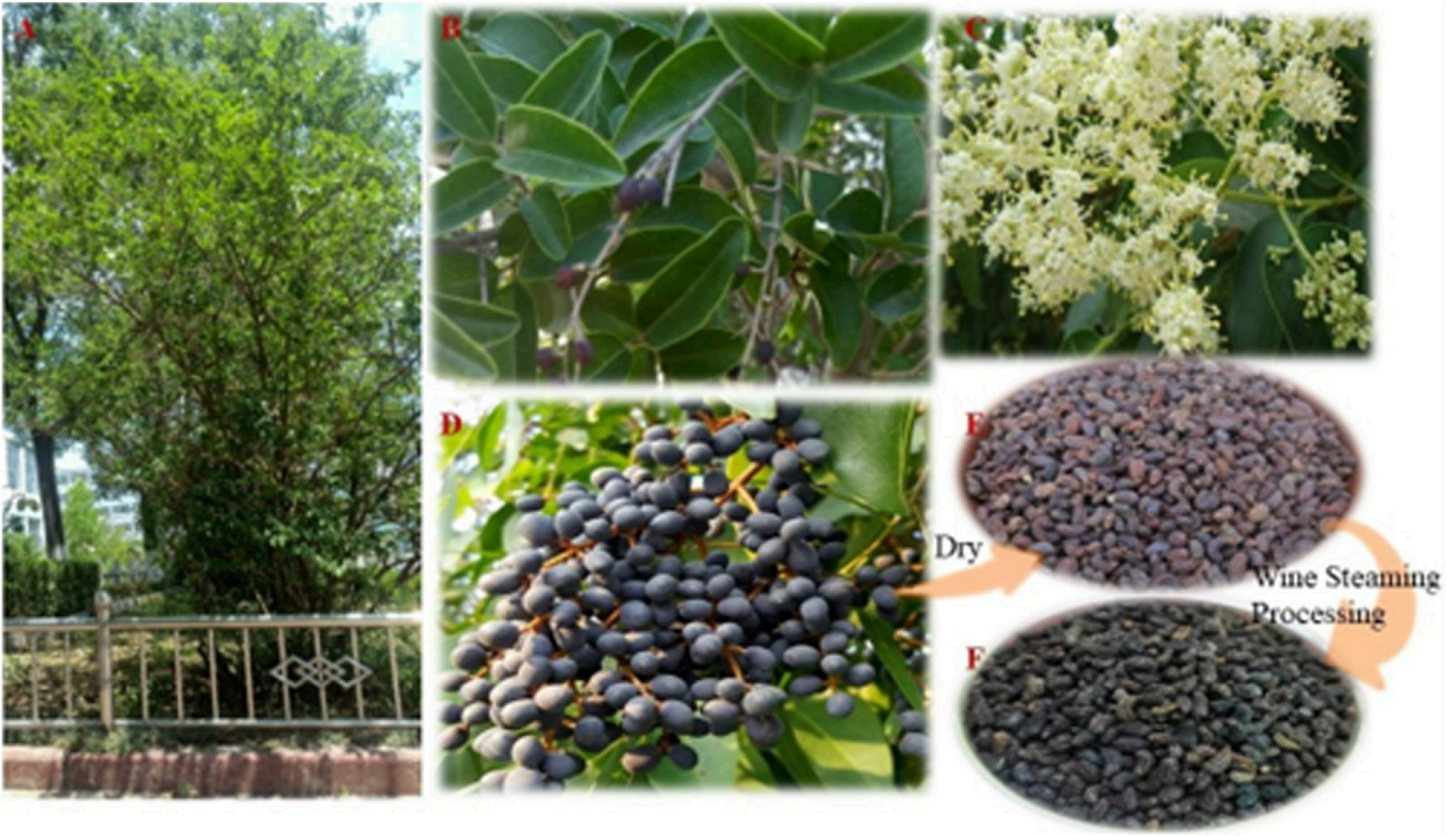
The whole plant **(A)**, leaf **(B)**, flower **(C)**, fresh ripe fruit **(D)**, desiccative ripe fruit **(E)**, and wine-processed product **(F)** of *Ligustrum lucidum* W.T. Aiton.

As shown in Figure 2, *L. lucidum* is a dramatic evergreen arbor tree, usually utilized as an ornamental tree for landscape. The hairless leaves are opposite, generally oval to oblong-oval or broadly elliptic, base rounded, thinly coriaceous, glossy dark green. The flowers are creamy or white and have a heavy fragrance. The inflorescences are broadly pyramid to cone in shape, 100–250 mm long and 200 mm wide. Young fruits are usually obovoid or ovoid in shape, and green in color. Ripe fruits are in large clusters of shiny purplish-black berries, which are 6–8 mm in diameter, endocarps thickly papery. Seeds are oval, 5–7 mm long, and 3 mm in diameter, typically one or two per fruit (Urcelay et al., 2019).

*Ligustrum lucidum* is mainly native to the southern half of China and Korea, whereas it was subsequently introduced for use as a garden and landscape ornamental plant in many other countries worldwide as well, such as Spain, Argentina, Italy, South Africa, Japan, Australia, the southern United States, and so on. In the late 19th century (Meiji Era), *L. lucidum* was first naturalized for use as an urban greening species in Japan. Whereas in Argentina, this herbal plant has been cultivated extensively since 1900 as hedging (Ren et al., 2020) *L. lucidum* is suitable for occurring in hot, full-sun and humid climates, where it grows fast, reaching 15 m or more of height. In China, this folk plant is distributed in at least 17 provinces, including Zhejiang, Jiangsu, Hunan, Sichuan, Anhui, Fujian, Gansu, Guangdong, Guangxi, Guizhou, Hainan, Henan, Hubei, Jiangxi, Shaanxi, Xizang, Yunnan (https://eol.org/pages/487035/articles#cite_note-12). As a consequence, there are abundant wild resources of *L. lucidum* worldwide.

## 3 Nutritional value and chemical composition

**FIGURE 3:**
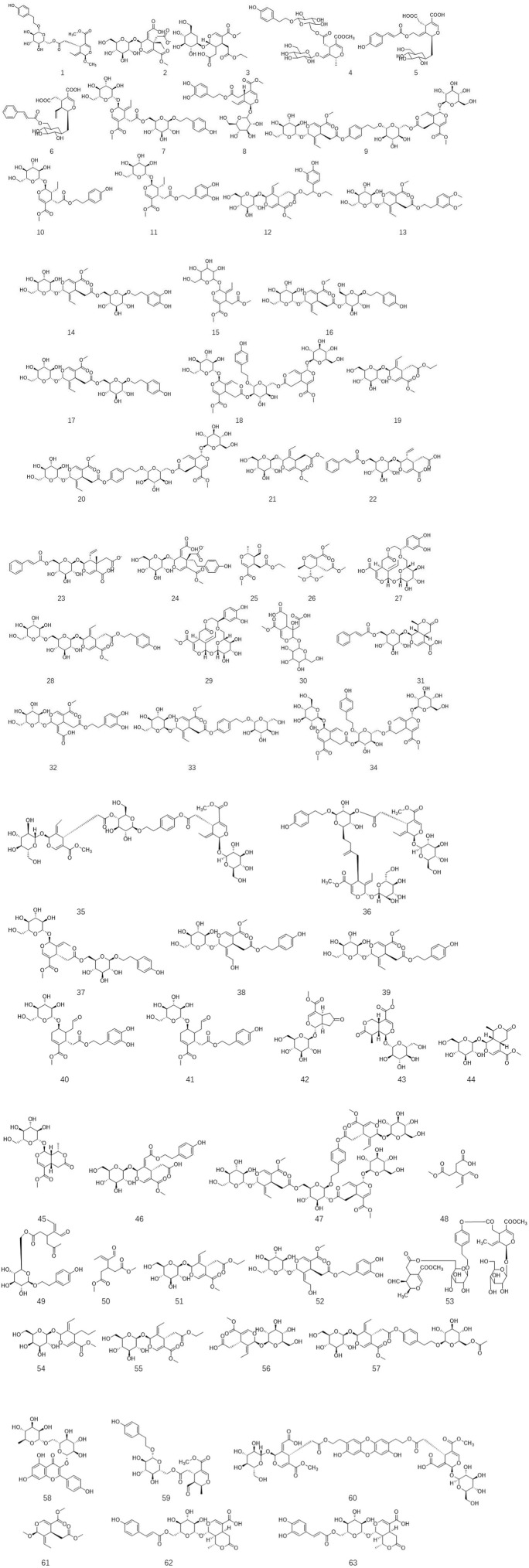
(Continued).

**FIGURE 4:**
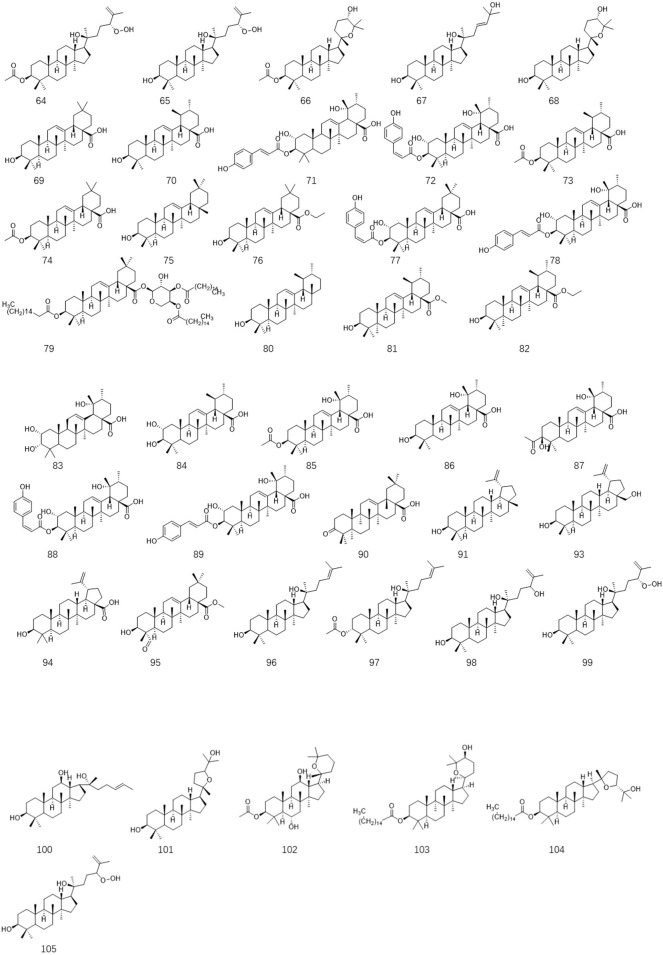
The chemical structures of triterpenoids isolated from *Ligustrum lucidum* W.T. Aiton.

**FIGURE 5:**
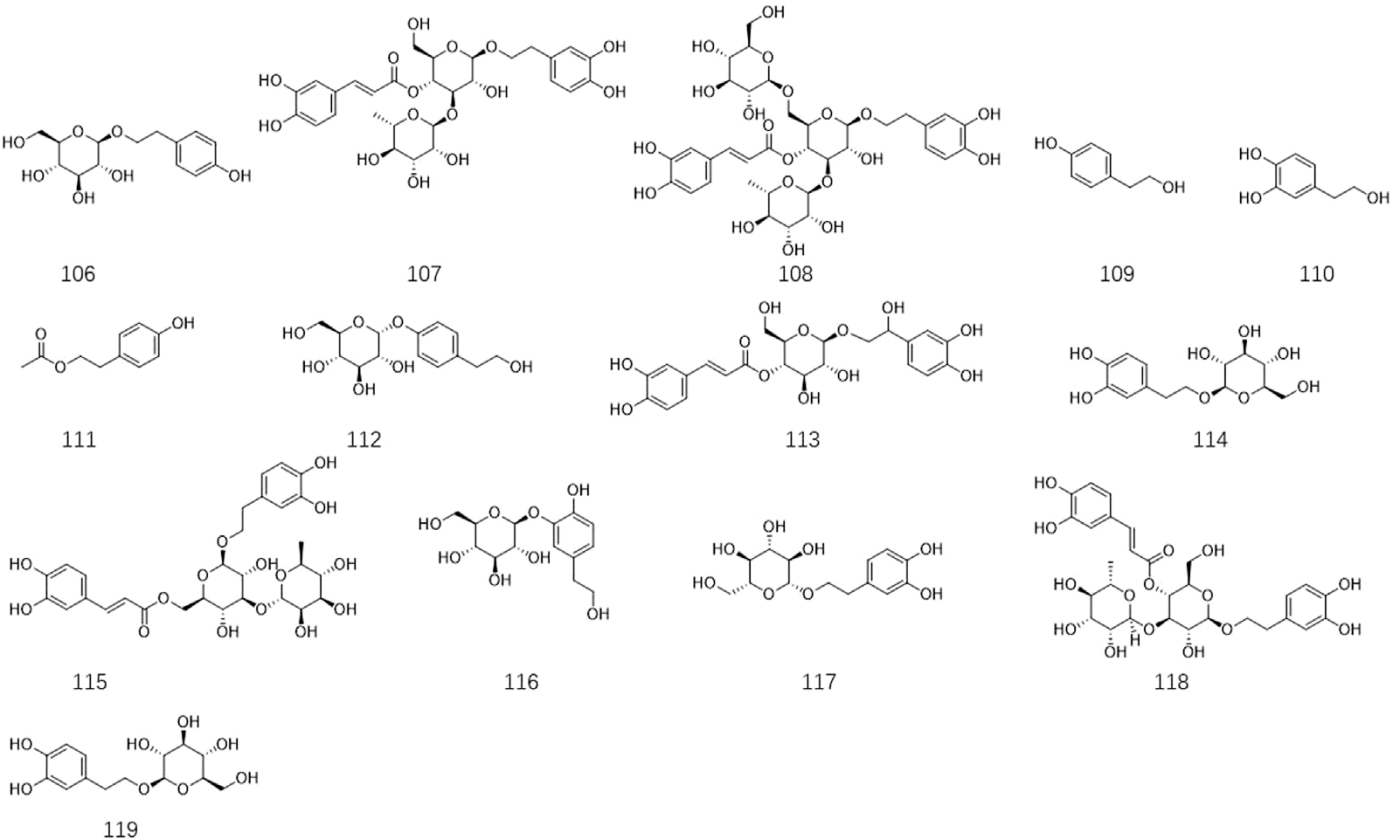
Phenylethanoid glycoside isolated from *Ligustrum lucidum* W.T. Aiton.

**FIGURE 6:**
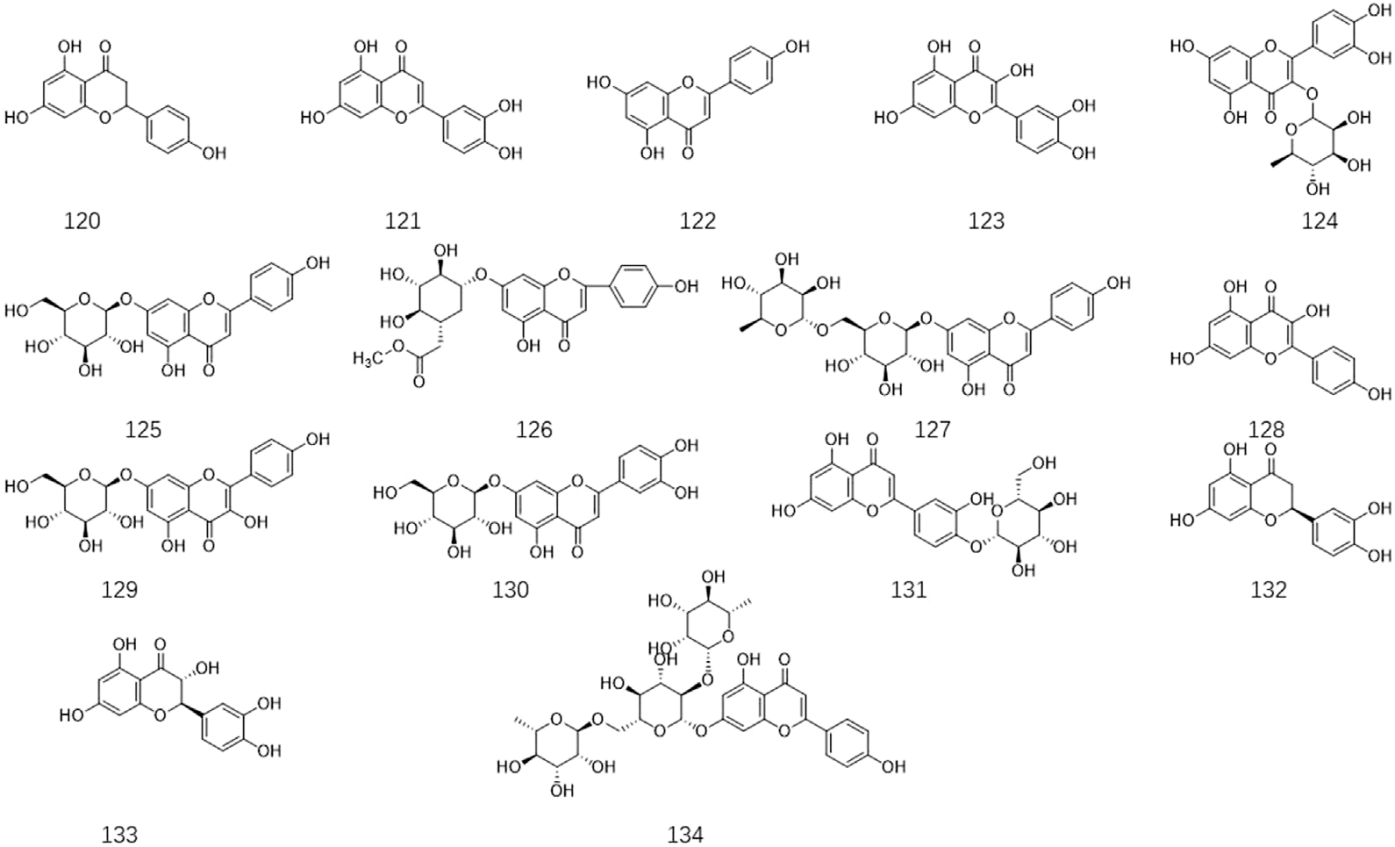
The chemical structures of flavonoids isolated from *Ligustrum lucidum* W.T. Aiton.

**FIGURE 7:**
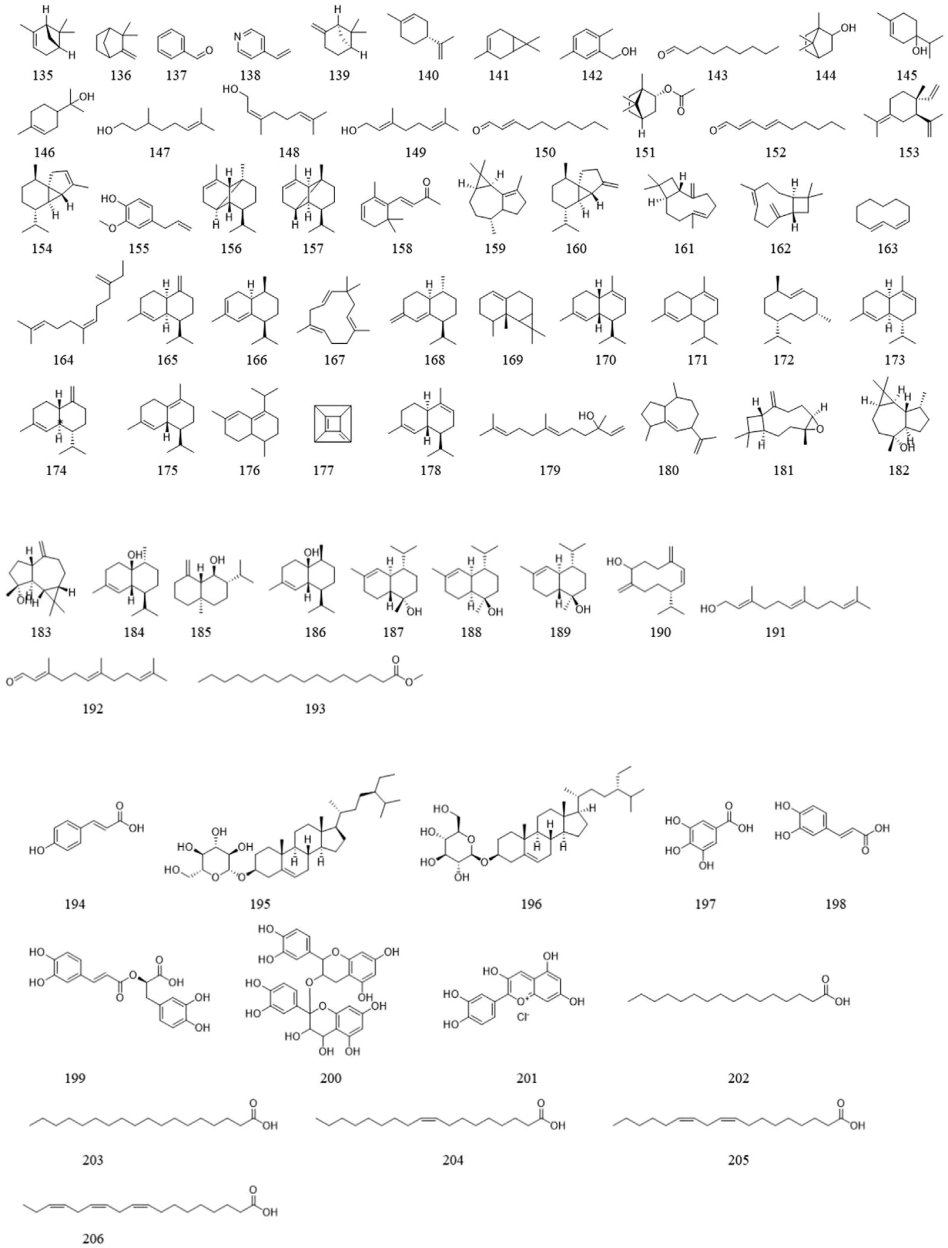
The chemical structures of other compounds isolated from *Ligustrum lucidum* W.T. Aiton.

*Ligustrum lucidum* is a good medicine food homology plant. Accumulating evidence has demonstrated that the components of *L. lucidum* are divided into nutritional components and bioactive components including iridoids (1–63), triterpenoids (64–105), phenylethanoid glycosides (106–119), flavonoids (120–134), volatile components (135–193) and polysaccharides. These chemical ingredients are summarized in Supplementary Table S1 and their structures are shown in Figures 3–7.

### 3.1 Nutritional components

The nutritional compounds of *L. lucidum* vary with geographic location, processing method, harvest time, and even extraction method (Li et al., 2021). The moisture content in *L. lucidum* is 40.3 g/kg, and the ash and solid content is 38.4 and 510.6 g/kg, respectively (Zhang et al., 2011). With the deepening of the research, it has been confirmed that the polysaccharides contained in the raw and processing products are different, and the polysaccharide content of the processing product has more advantages, which is about 8.7041–9.516 mg/g. *Ligustrum lucidum* polysaccharide is mainly composed of fucose, glucose, arabinose, and rhamnose with a molar ratio of 1.80:4.58:2.55:1.91 (Cao et al., 2019). The content of protein in *L. lucidum* is 80.3 g/kg, and the content of free amino acid is 2.9 mg/kg. Researchers identified about 21 amino acids (8 essential amino acids) in *L. lucidum*, with four major amino acids being aspartic acid, glutamic acid, alanine, and arginine (Zhao et al., 2016). In addition, it is rich in minerals including Potassium (K), Sodium (Na), Calcium (Ca), Magnesium (Mg), Phosphor (P), Zinc (Zn), and Iron (Fe) (Li et al., 2021). Hence, *L. lucidum* has considerable medical and nutritional value.

### 3.2 Iridoids

Iridoids, belonging to cyclopentane pyran monoterpenes, are acetal derivatives of iridodial. They usually react with sugar to form glycosides due to the unstable character of their C1-OH group. Based on the integrity of the cyclopentane unit, this large class of compounds has been divided into two types including iridoid glycosides and secoiridoid glycosides (Wang et al., 2020). Iridoids, especially secoiridoid glycosides, are widely prevalent in the plant kingdom, such as Caprifoliaceae, Nymphaeaceae, Gentianaceae, and Oleaceae. In secoiridoids, the C7–C8 in the parent nucleus of these active compounds is often broken to form a cleavage ring. *Ligustrum lucidum* is enriched with secoiridoids (Zhang et al., 2013). Up till now, over sixty-three secoiridoids have been isolated and purified from the leaves and fruits of *L. lucidum*. As described in Figure 2, all secoiridoids identified from L. lucidum have featured a β configuration of H-5. In 2013, the first 1-OCH3 substituent secoiridoid obtained from the plant kingdom, namely, ligulucidumoside A **1**, has been isolated and elucidated by spectroscopic methods and chemical analysis from the fruit of *L. lucidum* (Zhang et al., 2013). In 2018, three new secoiridoids, including nuezhenelenoliciside **4**, isojaslanceoside B **5**, and 6′-O-trans-cinnamoyl-secologanoside **6**, have been obtained and elucidated by comprehensive spectroscopic analysis. Among them, four featured a rare rearrangement product of secoiridoids, which existed the cleavage of a chemical bond between C-1 and O-2, then the reformation of a new iridoid ring between C-8 and O-2 (Qiu et al., 2018).

As a major class of chemical components in *L. lucidum*, these compounds have various biological activity. They have been demonstrated to prevent and treat many types of ailments such as pain, inflammation, hyperlipidemic, and hepatotoxicity as well as vision improvement effects (Liu H. et al., 2020; Wu et al., 2021). Specnuezhenide **7**, one of the most abundant components and its content used as the criteria to evaluate the quality of this crude herb, has been proven to possess strong biological effects, including regulating immunity, antivirus, anti-oxidation, and hepatoprotective activities. In 2001, He et al. first isolated lucidumosides C 12 and D 13, and **12** exhibited strong antioxidant effects (IC50 = 9.3 mM), which might be related to the number of phenolic hydroxyl groups (He et al., 2001). Two new secoiridoid glucosides, namely, iso-oleonuezhenide **18** and methyloleoside seven-ethyl ester 19 were isolated and assigned from the fruits of *L. lucidum*. This research has demonstrated that **18** induced the phosphorylation of ERK and CREB in primary cortical neurons in a dose-dependent and time-dependent manner, indicating its potential effect on neurons (Fu et al., 2010). Three new secoiridoid glycosides, ligulucisides A-C **22–24** as well as two new secoiridoids including liguluciridoids A **25** and B **26** were identified from the fruit of *L. lucidum* in 2018. Among these five compounds, **22, 24,** and **25** exhibited a better effect on anti-influenza A virus with the 50% inhibitory concentration (IC50) values of 16.5, 13.1, and 18.5 µM, respectively, compared to ribavirin (IC50 22.6 µM) (Pang et al., 2018).

### 3.3 Triterpenoids

Several investigations on *L. lucidum* have suggested that triterpenoids are another major component that has been isolated from this species. Until now, forty-two triterpenoids have been separated and characterized based on spectroscopic and chemical analysis from the fruits of *L. lucidum*. In 2008, five new dammarane triterpenes including 3β-acetyl-20S,24R-dammarane-25-ene-24-hydroperoxy-20-ol **64**, 20S,24R-dammarane-25-ene-24-hydroperoxy-3β, 20-diol **65**, 3β-acetyl-20S, 25-epoxydammarane-24a-ol **66**, 20S,25-epoxydammarane-3β, 24a-diol **67** and 20S-dammarane-23-ene-3β,20,25-triol **68** were isolated from *L. lucidum* for the first time (Xu et al., 2008). Oleanolic acid **69** and ursolic acid **70** are a pair of isomers that are obtained to be the ubiquitous triterpenoids in various medical plants. **69** and **70** illustrated from *L. lucidum* are the main effective constituents in this well-respected medicinal herb, which were used as the chemical markers for quality evaluation of *L. lucidum* preparations in the 2015 Chinese Pharmacopoeia (Cao et al., 2018b). Due to the thrilling reputation of these two compounds in bioactive efficacy, systematic pharmacology explorations of them have been carried out by different research groups. Accumulating evidence has indicated that these two compounds have exerted anti-aging, anti-inflammatory, antidiabetic, antioxidative, antitumor, antimutagenic, and anti-osteoporosis properties in *in-vivo* and *in-vitro* experimental studies (Zhang et al., 2007; Gao et al., 2009; Wu et al., 2010; Zhao et al., 2017; Cao et al., 2018a). Compounds 3-O-cis- p-coumaroyl maslinic acid (**71**) and 3-O-trans-p-coumaroyl maslinic acid (**72**) are two isomeric pentacyclic triterpenes found in *L. lucidum*, and 72 has specifically suppressed γ-secretase and decreased amyloid-beta levels, which suggesting used as a promising candidate for Alzheimer’s disease (AD) treatment (Luo et al., 2020). However, the bioactive properties of other triterpenoids remain unclear and need to be further explored. The structures of these compounds are shown in Figure 4.

### 3.4 Phenylethanoid glycosides

Phenylethanoid glycosides are typically presented in various plant kingdoms, which are essential phenolic components with anti-inflammatory, antibacterial, anti-tumor, neuro-protective, anti-viral, and other pharmacological activities. Therefore, phenylethanoid glycoside has captured considerable concern from scholars. At present, a total of fourteen phenylethanoid glycosides have been isolated and elucidated from the fruit and leaves of *L. lucidum* with unambiguous structures and significant bioactive characteristics. These compounds are shown in Supplementary Table S1 and Figure 5. Among the phenylethanoid glycosides, salidroside **106** is the characteristic chemical with broad pharmacological application prospect from *L. lucidum* and has generally been considered to be the quality control marker in wine-steamed *L. lucidum*, which should be quantified based on Chinese Pharmacopeia. Verbascoside **107** and Echinacoside **108** are the most frequently reported chemical components and have been proven to possess strong biological effects, such as anti-inflammatory, anti-hyperglycemic, anti-osteoporotic, liver protection, and anti-AD.

### 3.5 Flavonoids

Currently, fifteen flavonoids have been isolated and identified from the fruit, leaves, and flowers (Figure 6). There is much evidence to suggest that significant amounts of flavonoids are present in *L. lucidum* extracts (Jeong et al., 2016). It is noteworthy that six flavonoids were obtained from the flowers of *L. lucidum* for the first time in 2011, such as naringenin **120**, luteolin **121**, apigenin **122**, quercetin **123**, rutin **124**, and apigenin-7-O-glucoside **125** (Long et al., 2011). Previous research has indicated that ligustroflavone **134** is a natural flavonoid glycoside with excellent pharmacological properties, including anti-inflammatory, anti-fibrosis, antioxidant, anti-complementary, and anti-osteoporosis effects (Feng et al., 2019; Kang et al., 2021).

### 3.6 Polysaccharides

Plant polysaccharides have become a popular topic of research owing to their wide range of biological properties like antioxidant, immunomodulatory, and anti-tumor effects (Mukherjee et al., 2022). Research has uncovered a heteropolysaccharide extract from the fruits of *L. lucidum*, and the monosaccharide compositions of this plant’s polysaccharides are fucose, glucose, arabinose, and rhamnose (molar ratio of 1.80: 4.58: 2.55: 1.91) (Wang et al., 2003). Subsequently, Yin et al. obtained water-soluble polysaccharide extract with a good anticoagulant effect *in vitro* in the flowers of *L. lucidum*, which is composed of L-rhamnose, L-arabinose, D-xylose, D-glucose, and D-galactose in a molar ratio of 3.16: 2.46: 1.00: 7.27: 4.22 (Yin et al., 2017).

### 3.7 Others

In addition to the bioactive ingredients mentioned above, volatile components **(135–193)** and other compounds (194–206) have been extracted from *L. lucidum*. Relevant research has confirmed that possess certain antioxidant and antibacterial activities (Gao et al., 2022).

## 4 Biological and pharmacological activities

**FIGURE 8:**
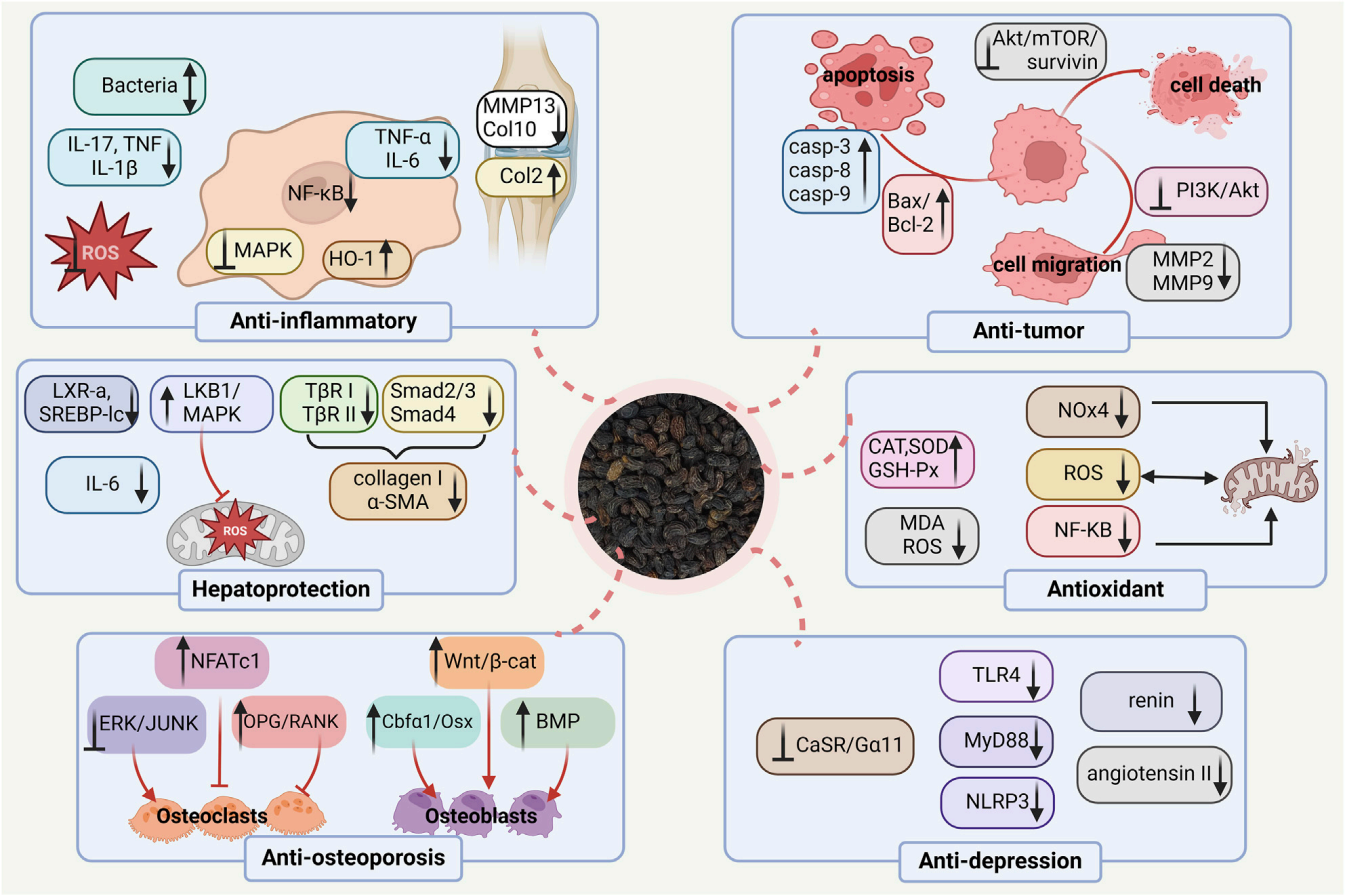
The main mechanisms of action of *Ligustrum lucidum* W.T. Aiton.

Herbal medicine, as the fundamental and vital part of many traditional medicines, has been gradually accepted for the prevention and treatment of different ailments worldwide, because of its multilevel function characteristics and remarkable efficacy with fewer adverse effects. *Ligustrum lucidum* is reputed to exert many strong pharmacological activities in *vivo* and *in vitro*, such as anti-osteoporosis, anti-fatigue, anti-oxidant, anti-tumor, anti-aging, anti-inflammatory, and so on. The pharmacological effects of *Ligustrum lucidum* have been summarized in Table 1 and Figure 8.

**TABLE 1:** Pharmacological effects of *Ligustrum lucidum* W.T. Aiton.

Activities	Extracts	Experimental model	Experimental dose	Effect	Mechanism	References
Anti-osteoporosis activity	Aqueous extract	OVX mice	1.75, 3.5, 7 g/kg	Inhibits oxidative stress, improves bone microstructure and osteoblast proliferation	Decreases MDA, increases SOD, BMD, BV/TV, Tb.N, Tb.Th, ALP and OPG, inhibits RANKL pathway	Wu et al. (2021)
		MC3T3-E1 cells	10^−1^–10^–5^ mg/mL			
	Ethanol extract	OVX rats with iron overload	700 mg/kg	Downregulates iron overload and promotes bone formation	Increases BMD, tb. Th, Tb. N and BV/TV, reduces tb. Sp, activates BMP-Smad pathway	Jiang et al. (2024)
	Ethanol extract	OVX mice	2, 4 g/kg	Improves bone microstructure, modulates bone and fat balance	Increases BMD, OPG and ALP, decreases tb. Sp, suppresses RANKL-RANK pathway, activate Wnt pathway	Qin et al. (2023)
		BMMSCs	0.01, 0.1 and 1 mg/mL			
	Aqueous extract	BMMSCs	50 and 100 μg/mL	Promotes the osteogenic differentiation	Stimulates proliferation, ALP and ARS, activate PI3K/AKT pathway	Kong et al. (2022)
	Aqueous extract	OVX rats	3.5 g/kg	Regulates the calcium balance and intestinal SCFAs production	Increases serum PINP and calcium, decreases CTx-I and urine calcium, increases calcium absorption	Chen et al. (2021)
	Aqueous extract	OVX rats; primary osteoblasts	3.5 g/kg	Attenuates the reduction in bone mineral density, strength and microstructure	Inhibits DKK1 and SOST overexpression, activate Wnt/β-catenin signaling	Liu et al. (2020b)
			1%–10% of medicated serum			
Antioxidant effect	Aqueous extract	OVX rats	3.5 g/kg	Inhibits oxidative stress response	Increases TAC and NO, decreases MDA and 8-OHdG, regulates Nox4/ROS/NF-КB pathway	Wang et al. (2017)
	Ethanol extract	BHT-induced rat	250, 500, 1,000 mg/kg	Decreases oxidative damage	Increases SOD, CAT and GPx, reduces MDA	Lin et al. (2007)
Hepatoprotective activity	Aqueous extract	CCl4-induced liver injury	100 mg/kg	Decreases oxidative damage	Decreases ROS, increase GSH, activates LKB1-AMPK pathway	Seo et al. (2017)
		HepG2 cells	100 μg/mL			
	Total glycosides	High fat diet-induced NAFLD mice	250 and 500 mg/kg	Regulates lipid metabolism and relieves lipid peroxidation injury	Decreased TG, TC, ALT, AST, SREBP-1c, LXR-α, and IL-6	Yang et al. (2015)
	Ligustroflavone	Liver fibrosis	5 and 20 mg/kg	Delays the formation of liver fibrosis	Decreases AST, ALT and MDA, increases Alb, GSH-Px and SOD, downregulates the TGF-β/Smad pathway	Kang et al. (2021)
		TGF-β1-stimulated LX-2	25 μmol/L			
Anti-inflammatory and analgesic activity	Aqueous extract	LPS-induced	100–500ug/mL	Alleviates neuroinflammation	Decreases NO, PGE2, TNF-α, IL-6, iNOS and COX-2, inhibit MAPK and NF-κB pathway	Kim et al. (2021)
		BV2 microglia				
		DSS-induced colitis mice	1 and 2 g/kg	Attenuates colitis, promotes the recovery from epithelial injury	Decreases ROS, CYP 450, CYP4A4 and P-Glycoprotein, rescues compromised microbiota	Yu et al. (2022)
		OA rat	100 μg/mL	Ameliorates pain response	Upregulates Col2 and Aggrecan, downregulates MMP13 and Runx2	Yan et al. (2018)
Anti-tumor effect	Methanol extract	Subcutaneous U87MG xenograft mice	50 and 100 mg/kg	Inhibits glioma tumor growth, induces apoptotic cell death	Downregulate Akt, mTOR and survivin, regulate the Akt/mTOR/survivin pathway	Jeong et al. (2011)
			1–30 mg/mL			
	Aqueous extract	Bel-7402 cell	100–400 μg/mL	Inhibits proliferation, induces apoptosis ansenescence	Activate caspase-3, -8 and -9, upregulate p21, downregulate p-RB	Hu et al. (2014)
	Ethanol extract	HCC mice	1.5, 3, 5 g/kg	Inhibits tumor growth, migration and invasion, promots apoptosis and cell cycle arrest	Increases Bax, TIMP2; decreases Bcl-2, CytC, caspase-3, Ki67, Cyclin D1, p21, MMP2, MMP9, p-PI3K, PI3K, Akt and p-Akt	Tian et al. (2019)
		Bel-7402 and Huh-7 cell lines	25, 50, 75 mg/mL			
Anti-depressive effects	Phenol glycosides extract	Depressive-like mice	75, 225 mg/kg	Improves depressive symptoms and neuroinflammation	Decreases IL-1β, Rantes, MCP-1, TLR4, MyD88, NLRP3, CaSR, Gα11, renin and Ang II, increases	Feng et al. (2020)
					1-OHase	
Immunomodulatory activity	CO_2_ extract	Piglet immune cells	5, 25, 50 or 100 mg/L	Enhances the prolifer-ative activity of blood lymphocytes	Increases CD^4+^ CD^8+^, IL-2, IFN-γ, TNF-α and NO, decreases IL-4 and IL-10	Wang et al. (2012)

### 4.1 Anti-osteoporotic activity

Osteoporosis, a refractory disease, becomes a kind of universal occurrence in older people worldwide, which can lead to decreased life functioning and quality of life to a considerable extent. The prevalence of this skeletal ailment has posed severe public health and economic challenges to our society (Ensrud and Crandall, 2024). *Ligustrum lucidum* has traditionally been used for the treatment of bone diseases in TCM for thousands of years. As one of the liver and kidney-tonifying herbs and consistency in the TCM theory of “kidney governing bones,” L*. lucidum* is especially applied to prevent and treat postmenopausal osteoporosis (PMOP) (Chen et al., 2021; Qin et al., 2023). It also exerts anti-osteoporotic effects in diabetes-induced osteoporosis (Feng et al., 2019), senile osteoporosis (Li et al., 2019), and oxidative stress-related osteoporosis (Wu et al., 2021). *Ligustrum lucidum* has been reported to downregulate iron overload and promote bone formation in ovariectomized (OVX) rat models through the BMP-Smad pathway after continuous administration for almost 2 months (Jiang et al., 2024).

Appropriate control of short-chain fatty acids (SCFAs) production and calcium metabolism is a new avenue in the prevention of osteoporosis (Lucas et al., 2018). In 2021, Chen et al. assessed the effects of *L. lucidum* aqueous extract on SCFAs production, calcium balance, and bone homeostasis in the model of OVX rats (Chen et al., 2021). The extract (3.5 g/kg) was daily and orally administered to ovariectomized rats for 14 weeks. Compared with the control group that received appropriate amounts of saline alone. The level of calcium-sensing receptor (CaSR) expression was decreased, and surprisingly, the extract effectively increased SCFAs levels and calcium absorption. In addition, the extract has been shown to suppress the decline of the bone microstructure, bone strength, and various bone material properties. Thus, the aqueous extract may preserve bone quality via prevention against calcium loss and regulation of the SCFAs production in ovariectomized rats. Additionally, *L. lucidum* ethanol extract and its two primary compounds, ursolic acid and oleanolic acid, significantly inhibit osteoclast differentiation and bone resorption of RAW264.7 murine monocyte/macrophage cells through receptor activator of nuclear factor КB ligand (NF-КB) signaling pathways (Xu et al., 2016). However, this study had apparent limitations one dose of the extract was applied, and thus the information on the dose-dependent activity was limited.

### 4.2 Antioxidative activity

As can be seen from the above, antioxidation is the underlying mechanism of *L. lucidum* in treating many diseases. For example, oxidative stress is considered to play a contributory role in the imbalance between bone resorption and formation, and thus this has received increasing prominence for the elucidation of post-menopausal osteoporosis. In ovariectomized rats, oral administration of *L. lucidum* aqueous extract (3.5 g/kg, i. g.) prevented the accumulation of free radicals and NF-КB activation, and thus decreased oxidative damage and improved bone microstructure and mineral density, which indicated the correlation between antioxidation and anti-osteoporotic effects (Wang et al., 2017). However, a single-dose study was included in this trial thus necessitating the dose-dependent study. In 2007, Lin et al. investigated the antioxidant activities of *L. lucidum* ethanol extract and its effects on butylated hydroxytoluene (BHT)-induced oxidative stress in Male Wistar rats. The result revealed that the extract could effectively inhibit the acute BHT-deduced oxidative stress through the decline in serum glutamic pyruvic transaminase, glutamic oxaloacetic transaminase, alkaline phosphatase, and lactate dehydrogenase as well as the upregulation of antioxidant enzymes in lung, liver and kidney (Lin et al., 2007). However, no positive control was included in this trial.

### 4.3 Liver protective activity

For centuries, *L. lucidum* has been commonly used as a traditional health food to nourish and detoxify the liver worldwide including in China, Korea, and so on (Yao et al., 2013). According to Seo’s study, oral administration of 100 mg/kg *L lucidum* aqueous extract dramatically attenuated the activities of alanine aminotransferase and oxidative stress in the liver, showing a protective effect on CCl4-stimulated liver injury in C57BL/6 male mice. Meanwhile, *in vitro* experiments showed that the extract (100 μg/mL) inhibited arachidonic acid + iron-induced ROS generation, GSH depletion, and mitochondrial dysfunction via the AMPK pathway in HepG2 cells (Seo et al., 2017). However, these investigations lacked appropriate positive controls. CCl4 is one of the most common xenobiotics for induced-liver injury and the injury can be strongly attacked by Ligustroflavone (5 mg/kg and 20 mg/kg, i. p.). An *in vitro* study (25 μmol/L) found that the fundamental mechanism of antifibrotic effects was through down-regulating the TGF-β/Smad signaling pathway in the human hepatic stellate cell line (LX-2) (Kang et al., 2021). However, this study was conducted without the use of a positive control.

### 4.4 Anti-inflammatory and analgesic activity

It was reported that the use of *L. lucidum* water extract possessed an analgesic effect on the osteoarthritis (OA) rat model. A related study established the rat OA model through intra-articular (IA) injection of mono-iodoacetate. After IA administration (100 μg/mL), the extract effectively ameliorated joint pain response through increasing heat pain sensitivity and thresholds of mechanical allodynia as well as spontaneous activity, demonstrating the analgesic function of *L. lucidum* (Yan et al., 2018). Nevertheless, the major shortage of this study was only conducted with a single dose.

### 4.5 Anti-tumor activity

The wide application of health-strengthening herbs in cancer treatment has given rise to growing research interests all over the world. *Ligustrum lucidum* has been demonstrated to exert its characteristic antitumor potential. One study showed that *L. lucidum* extract inhibited glioma tumor growth *in vivo* and induced glioma cell death *in vitro*, the underlying mechanism involved in the regulation of the Akt/mammalian target of rapamycin (mTOR)/survivin pathway. Therefore, *L. lucidum* might serve as a potential drug candidate for malignant human gliomas (Jeong et al., 2011). Yet the study did not illustrate the major active component of the extracts inducing suppression of glioma tumor growth. Hu et al. found that *L. lucidum* aqueous extract suppressed the proliferation of human hepatocellular carcinoma Bel-7402 cells in a dose-dependent and time-dependent manner (50–800 μg/mL for 24 h, 48 h, and 72 h). The extract also had a pro-apoptotic ability in the Bel-7402 cells, by causing huge and flat morphologic cellular change, and blocking the G0/G1 cell cycle, accompanied by sensitization of caspases. To further illustrate the mitochondrial transformation in apoptosis induced by the aqueous extract, it was demonstrated that *L. lucidum* contributed to the Bel-7402 cell apoptosis and cell senescence through upregulation of p21 and downregulation of RB phosphorylation (Hu et al., 2014). In addition, Tian et al. confirmed that the ethanol extract of *L. lucidum* leaves possessed excellent anti-tumor effects on hepatocellular carcinoma (HCC) *in vitro* and *in vivo*. The results showed that the extract effectively promoted the apoptosis of Bel-7402 and Huh-7 cells by regulating the activity of caspase-3, as well as the expressions of B cell lymphoma 2 (Bcl-2), Bcl-2 associated X (Bax) and Cytochrome-C (CytC). It was demonstrated that the extract had a markedly inhibitory effect on cell migration and invasion. In addition, it strongly decreased the expressions of matrix metalloproteinase2 (MMP2) and MMP9 and enhanced the expression of tissue inhibitors of metalloproteinases two in Bel-7402 and Huh-7 cells. The ethanol extract of *L. lucidum* leaves mediated by the suppression of Phosphoinositide 3-kinase (PI3K)/Akt pathway was closely associated with DNA de-methylation of PTEN. In further *in vivo* studies, the extract suppressed the tumor growth of hepatocellular carcinoma (Tian et al., 2019). However, further researches are needed to determine which components are involved in *L. lucidum*-induced antitumor activities.

### 4.6 Anti-depressive effects

Depression is the most common of the severe neuropsychiatric illness and has been identified as the critical cause of disability worldwide (Beurel et al., 2020). Inflammatory processes in the central nervous system are associated with the pathogenesis of depression (Leng et al., 2018). Of noted, the phenol glycosides from *L. lucidum* (75 mg/kg and 225 mg/kg, i. g.) were elucidated to have beneficial ameliorative effects on lipopolysaccharide (LPS)-triggered depressive-like performance in mice, which might be dependent on repressing neuroinflammation in the hypothalamus that was mediated by abrogation the activation of microglia and the release of inflammatory cytokines by regulation on f toll-like receptor-4 (TLR4) signaling pathway (Feng et al., 2020). Nonetheless, the study lacked positive control.

### 4.7 Immunomodulatory activity

The CO_2_ supercritical extract from *L. lucidum* (5, 25, 50, and 100 mg/L) was found to promote immune responses through stimulating lymphocyte proliferation as well as upregulation of the lymphocyte populations of CD4^+^ CD8^−^ and CD4^+^ CD8^+^. Furthermore, the extract regulated the expression of Th1- and Th2-related cytokines, enhanced the levels of IFN-γ, IL-2, and TNF-α in helper T-cells (Th)1, decreased the release of IL-4 and IL-10 in Th2, and stimulated the secretion of NO (Wang et al., 2012).

## 5 Applications

### 5.1 Medicine field

*Ligustrum lucidum* is listed as a “Top grade” drug in Shen Nong’s Herbal (Dong Han Dynasty, A.D. 25–220). According to TCM, women, winter, and quiet characters belong to yin in China. From the name of *L. lucidum*, we can understand how the ancients interpreted its hallmarks and nature. Traditionally, the leaves and fruits are obtained, dried, and used in clinical practice for several purposes, such as preventing aging and hair graying, improving vision, alleviating the soreness and weakness of the waist and knees, and strengthening human energy in its native and introduced areas. The theoretical basis of TCM is kidney domains bone and produces marrow, as a general tonic herb, the dried mature fruits are documented in the version of 2020 Chinese pharmacopeia, possessing the effects to invigorate muscles and bones, supplement the kidney and liver, nourish yin, and clear vision. In traditional Japanese and Swedish medicine, *L. lucidum* has been used to treat osteoporosis (Wegiel and Persson, 2010). It has also been commonly applied in traditional Korean medicine to detoxify the liver and kidneys (Kim et al., 2021).

#### 5.1.1 Herb pairs application

Herbal formulae are the predominant pattern of traditional clinical practice. Herb pairs are the most essential unit and the simplest form of TCM prescription. As a bridge between a single herb and a complex formulation, herb pairs amass the experience of veteran TCM doctors, greatly interpret the traits of the “gregarious utilization” of TCM, and perfectly embody the connotation of holistic therapeutic theory and syndrome differentiation treatment (Wang et al., 2022). Herb drugs usually consist of a unique combination of two or three relatively fixed herbs, which embody a centralized representative of herbal compatibility according to their characteristics, such as strengthening treatment function and attenuating virulence through potentially synergistic herb interactions compared to single herb (Han et al., 2020). *Ligustrum lucidum* not only can treat diseases with single medicine but also can be widely used to treatment of immunodeficiency, bone marrow suppression, and cancer when it is combined with other drugs. The herb pairs compatibility of *L. lucidum* are summarized in Table 2.

**TABLE 2:** Some herb pairs associated with *Ligustrum lucidum* W.T. Aiton.

Composition of herb pairs	Traditional and clinical uses	References
*Ligustrum lucidum* fruit, *Epimedium brevicornu leaf*	Tonify yin and yang, invigorate the essence and strengthen bones, treat kidney-yang deficiency syndrome of asthma, prevent osteoporosis and orthopedic-related diseases	Ma et al. (2020)
*Ligustrum lucidum* fruit, *Epimedium brevicornu* leaf, *Psoralea corylifolia* L. fruit	Improve bone mineral density in post-menopausal women suffering from osteopenia	Knight and Baune (2018)
*Ligustrum lucidum* fruit, Astragalus membranaceus root	Treat immunodeficiency diseases	Zhao et al. (2022)
*Ligustrum lucidum* fruit, *Panax ginseng* C. A. Meyer root	Treat bone marrow suppression after chemotherapy, invigorate qi tonify blood, and nourish yin	Han et al. (2020)
*Ligustrum lucidum* fruit, Acanthopanax senticosus root and rhizome	Treat myelosuppression induced by chemotherapy	Wang et al. (2019)
*Ligustrum lucidum* fruit, Ganoderma lucidum mushroom	Tonify energy and reinforce deficiency, improve the quality of life and emotional wellbeing among non-small cell lung cancer patients	Liu et al. (2020b)
*Ligustrum lucidum* fruit, *Polygonum cuspidatum* Sieb. et Zucc. root and rhizome	Treat acute gouty arthritis	Du et al. (2022)

#### 5.1.2 Prescription application

There are hundreds of *L. lucidum*-related prescriptions in the clinic, as shown in Table 3. Erzhi Pill (EZP), a classic TCM prescription used for the treatment of Xiaoke disease and turbid urine, was initially documented in Fu Shou Jing Fang by Wu MinJi in China during the Ming Dynasty (A.D.1530). EZP is a simple combination of only two medicinal herbs, which consists of equal amounts (at a ratio 1:1) of *Ligustri Lucidi* and Ecliptae Herba. This formulation has been used to prevent and treat various kidney diseases by tonifying the body’s essential fluid, enhancing tendons and bones, and arresting hemorrhage. Nowadays, EZP is the most frequently mentioned as an anti-aging, hepatoprotective, promoting hematopoietic, and anti-oxidant agent for menopausal symptoms in clinical application in Taiwan and China (Chen et al., 2011; Cheng et al., 2011; Xu et al., 2012). EZP has shown marked effects in the treatment of diabetic cardiomyopathy (Peng et al., 2022), benign prostatic hyperplasia (Tao et al., 2022), liver injury (Zhao et al., 2018), AD (Xie et al., 2021), osteoporosis (Zhong et al., 2021), rheumatoid arthritis (RA) (Li et al., 2020), fatty liver (Huang et al., 2020), and aging-associated symptoms (Feng et al., 2021).

**TABLE 3:** Some classical prescriptions associated with *Ligustrum lucidum* W.T. Aiton.

Preparation name	Composition	Formulation	Traditional and clinical uses	References
Erzhi pill	*Ligustrum lucidum* fruit, *Eclipta prostrata* herb	Pill	Tonify the kidney, nourish yin and clear away heat; clinically used to treat various kidney diseases and liver injuries	Fu shou jing fang
				《扶寿精方》
Kangfu wan	*Rehmannia glutinosa* root, *Ligustrum lucidum* fruit, *Angelica sinensis* root, *Pseudostellaria heterophylla* root, *Dipsacus asper* semen, *Cuscuta australis* semen, *Dioscorea opposite* rhizome, *Schisandra chinensis* fruit, *Polygonum multiflorum* stem, Lycium chinense cortex, margaritifera concha, Talci pulvis	Pill	Tonifying kidney, nourishing blood and calming nerves; clinically used to treat various diseases, such as treat many diseases such as insomnia, forgetfulness, headaches, dizziness, and night sweating	https://www.ncmi.cn/index.html
Shiquan buyin tang	*Asparagus cochinchinensis* root, *Ophiopogon japonicus* root, *Ligustrum lucidum* fruit, *Eclipta prostrata* herb, *Paeonia lactiflora* root, *Glycyrrhiza uralensis* root and rhizome, *Imperata cylindrica* rhizome, *Nelumbo nucifera* stem, *Salvia miltiorrhiza* root and rhizome, *Cyperus rotundus* rhizome	Decoction	Now used in the treatment of menstrual disorders, tinnitus, and dizziness	https://www.ncmi.cn/index.html
Zhenqi fuzheng capsule	*Astragalus membranaceus* root, *Ligustrum lucidum* fruit	Capsule and pill	Improvement of immunity and promotion the recovery of normal functions after surgical operations	Xue et al. (2021)
Yangzheng xiaoji capsule	*Astragalus membranaceus* root, *Ligustrum lucidum* fruit, *Panax ginseng* root and rhizome, *Curcuma phaeocaulis* rhizome, *Ganoderma lucidum* mushroom, *Gynostemma pentaphyllum* root, *Atractylodes macrocephala* rhizome, *Scutellaria barbata* herb, *Hedyotis diffusa* herb, poria cocos, *Eupolyphaga sinensis* Walker, *Duchesnea indica* herb, *Solanum lyratum* herb, *Artemisia scoparia* herb, Cynanchum paniculatum root	Capsule	Commonly used for various solid tumors	Chinese pharmacopoeia, 2020
Kanggu Zengsheng Wan	*Rehmannia glutinosa* root, *Cistanche deserticola* rhizome, *Ligustrum lucidum* fruit, *Epimedium brevicornu* leaf, *Spatholobus suberectus* stem, *Raphanus sativus* semen, *Drynaria bonii* rhizome, *Achyranthes bidentata* root	Pill	Tonifying kindey, strengthening tendon and bone, invigorating the blood circulation, relieving pain; used in the treatment of osteoarthritis	Chinese pharmacopoeia, 2020
BushenYijingWan	*Ligustrum lucidum* fruit, *Cuscuta australis* semen, *Eclipta prostrata* herb, *Schisandra sphenanthera* fruit, *Morus alba* root, *Rubus chingii* fruit, *Cistanche deserticola* herb, *Rehmannia glutinosa* root	Pill	Tonifying kidney, filling marrow, and nourishing the essence and blood; used for kidney-yin deficiency, waist and knee weakness, and spermatorrhea	Chinese pharmacopoeia, 2020
Guxian pian	*Rehmannia glutinosa* root, *Lycium barbarum* fruit, *Ligustrum lucidum* fruit, *Glycine* max semen, *Cuscuta australis* semen, *Drynaria bonii* rhizome, *Curculigo orchioides* rhizome, *Achyranthes bidentata* root, *Stephania tetrandra* root	Tablet	Strengthen tendons and muscles, dredging collaterals, and relieving pain; used for treatment arthralgia, numbness of hand or foot, and hyperosteogeny	Chinese pharmacopoeia, 2020
Ningshen Buxin pian	*Salvia miltiorrhiza* root and rhizome, *Rehmannia glutinosa* root, *Ligustrum lucidum* fruit, *Eclipta prostrata* herb, *Acorus tatarinowii* rhizome, *Polygonum multiflorum* stem, *Albizia julibrissin* cortex, *Schisandra chinensis* fruit	Tablet	Activating blood, calming and soothing the nerves, filling marrow; used in treatment of kidney and blood deficiency associated with palpitations, tinnitus and dizziness	Chinese pharmacopoeia, 2020
Yishenling keli	*Lycium barbarum* fruit, *Ligustrum lucidum* fruit, *Aconitum carmichaelii* root, *Euryale ferox* semen, *Plantago asiatica* semen, *Psoralea abbottii* fruit, *Rubus chingii* fruit, *Schisandra sphenanthera* fruit, *Morus alba* fruit, *Astragalus complanatus* semen, *Allium tuberosum* semen, *Epimedium brevicornu* leaf, *Rosa laevigata* fruit	Pill	Warming yang, tonifying kidney; used for treatment kidney-yang deficiency syndrome, manifested as erectile dysfunction, premature ejaculation and spermatorrhea	Chinese pharmacopoeia, 2020
Shengxuebao keli	*Polygonum multiflorum* root, *Ligustrum lucidum* fruit, *Morus alba* fruit, *Eclipta prostrata* herb, *Paeonia lactiflora* root, *Astragalus membranaceus* root, *Cibotium barometz* rhizome	Pill	Tonifying the liver and kidney, nourishing qi and activating blood; commonly used for liver-kidney yang deficiency and qi-blood deficiency syndrome	Chinese pharmacopoeia, 2020
Huamoyan keli	*Prunella vulgaris* spike, *Ligustrum lucidum* fruit, *Ilex cornuta* leaf, *Astragalus membranaceus* root, *Stephania tetrandra* root, *Coix lacryma-jobi* semen, *Smilax glabra* rhizome, *Luffa cylindrica* fruit, *Lycopus lucidus* herb, *Salvia miltiorrhiza* root and rhizome, *Angelica sinensis* root, *Cyathula officinalis* root, herba Siegesbeckiae	Pill	Clearing away heat and expelling dampness, activating blood and dredging collaterals; used for treatment of blood stasis blocking collateral	Chinese pharmacopoeia, 2020
Kanggu Zengsheng capsule	*Rehmannia glutinosa* root, *Cistanche deserticola* herb, *Cibotium barometz* rhizome, *Ligustrum lucidum* fruit, *Epimedium brevicornu* leaf, *Spatholobus suberectus* stem, *Raphanus sativus* semen, *Drynaria roosii* rhizome, *Achyranthes bidentata* root	Capsule	Tonifying kidney, strengthening tendons and muscles, as well as activating blood and relieving pain; used for treatment arthralgia, numbness of hand or foot, and hyperosteogeny	Chinese pharmacopoeia, 2020
Weidakang koufuye	*Acanthopanax senticosus* root and rhizome, *Astragalus membranaceus* root, *Citrus reticulata* fruit peel, *Rehmannia glutinosa* root, *Ligustrum lucidum* fruit, *Aconitum carmichaelii* root, *Epimedium brevicornu* leaf	Liquid pharmaceutical Preparations	Strengthening healthy qi and root, tonifying kidney and calming the nerves; commonly used for treatment dyspnea, fatigue, nausea, poor appetite, drowsiness, sleep disturbances caused by kidney essence deficiency	Chinese pharmacopoeia, 2020
Yening Tangjiang	*Albizia julibrissin* cortex, *Ganoderma lucidum* mushroom, *Polygonum multiflorum* stem, *Ziziphus jujuba* fruit, *Ligustrum lucidum* fruit, *Glycyrrhiza uralensis* root and rhizome	Liquid pharmaceutical Preparations	Nourishing blood and calming nerves, now used in the treatment of the syndrome of palpitation, insomnia and dreaminess associated with heart-blood deficiency	Chinese pharmacopoeia, 2020
Qiwei xiaoyan Tang	*Rheum palmatum* root and rhizome, *Astragalus membranaceus* root, *Wikstroemia indica* root, *Ligustrum lucidum* fruit, poria, *Glycyrrhiza uralensis* root	Decoction	Treat bronchitis, tonsillitis, hepatitis, diarrhea, hemorrhoids, enteritis and infection	He et al. (2013)
Zhenqing recipe	*Ligustrum lucidum* fruit, *Eclipta prostrata* herb, *Dioscorea opposite* rhizome	Decoction	Now used in the improvement diabetic nephropathy	Wen et al. (2012)

Another famous folk formula is the Zhenqi Fuzheng formula (ZQFZ), composed of Ligustri Lucidi Fructus and Astragali Radix (1:2, w/w). According to the qi-blood theory of TCM, qi is the commander of blood, and blood is the mother of qi. Qi and blood are mutually dependent. Qi deficiency or blood-deficiency may cause all kinds of diseases. ZQFZ is the role of supplementing-Qi and enriching blood and is usually utilized clinically to improve immunity, increase leukocytes, protect bone marrow and adrenal cortex (Shi et al., 2011; Miao et al., 2018). Complementary and alternative medicine has gradually gained popularity in the world over the last decades. As an adjuvant therapy, TCM preparation decreases dramatically adverse effects induced by surgery, radiotherapy, and chemotherapy, and acts synergistically with them. ZQFZ is also used in clinics to treat a variety of cancers, and is widely used together with or after these therapies to promote the recovery of body functions, prolong the survival time of cancer patients, and improve their quality of life. Accumulation research indicated that the curative effect is remarkable (Li et al., 2020; Meng et al., 2022).

Moreover, there are some undocumented *L. lucidum*-related formulae frequently utilized by traditional Chinese doctors like Qiwei Xiaoyan Tang (He et al., 2013), Shuangdi Shouzhen Tablet (Fang et al., 2021), Zhenqing recipe (Wen et al., 2012; Song et al., 2020) and so on.

### 5.2 Food application

As a compelling tonic herb, the fruit of *L. lucidum* is not just used in treatment for ailments but also as health food owing to its excellent liver tonic, kidney tonic, and hair blackening (Guo et al., 2008).

#### 5.2.1 Functional food

Based on its rich efficacy and high safety profile, *L. lucidum* has been made into a variety of functional foods, mostly with immune regulation, liver function protection, anti-fatigue, and hypoglycemic effects. The products mainly include tablets, capsules, granules oral liquid, wine, and tea preparation, as shown in Table 4 (http://ypzsx.gsxt.gov.cn/specialfood/#/food). They are mostly in traditional preparations and need to be further innovated.

**TABLE 4:** Healthcare products based on *L. lucidum*.

Product name	Component content (Per 100 g content)	Drug efficacy	Approval number
Yiling Brand Ginseng and L. lucidum Ganoderma Capsules	Crude polysaccharide 3.2 g, total saponin 0.9 g	Immunity enhancement	G20220034
Yuzhilin Brand Colla Corii Asini L. lucidum Soft Capsule	Protein 15g	Immunity enhancement	G20230682
Heng Wei Brand Astragalus and L. lucidum Rhodiola Rosea Capsules	Crude polysaccharide 1.4 g, salidroside 0.337 g	Immunity enhancement	G20210152
Zhangzisong Brand L. lucidum Velvet antler Wine	Crude polysaccharide 0.0274 g	Immunity enhancement	G20130403
Yi-Tai Gold American Ginseng, L. lucidum, and Schisandra Oral Liquid	Total Saponin 0.053 g	Immunity enhancement	G20110575
Zhu Li Brand Astragalus and L. lucidum Tablets	Crude polysaccharide 4 g, adenosine 0.012 g	Immunity enhancement	G20200612
Songzhen Brand Rehmannia glutinosa L. lucidum Capsules	Crude polysaccharide 0.31 g, total flavonoids 0.48 g	lowering blood glucose	G20080293
Baibang Brand L. lucidum Polygonatum odoratum Capsules	Total Saponin 1 g, total Flavonoid 0.6 g	lowering blood glucose	G20100127
JIN Brand Cordyceps Ginseng, Horse Antler, Dendrobium Officinale, Chasteberry Capsule (Men’s Type)	Adenosine 0.0035 g, total Saponin 0.3 g	relieving physical fatigue	G20160046
Yiheng Hovenia dulcis L. lucidum Schisandra capsules	Total flavonoids 0.035 g, schisanol A 0.25 g	Protection from chemical liver injury	G20200432
Ao-dong Brand L. lucidum Hawthorn Tablets	Total saponin 0.709 g, total flavonoid 0.771 g, Emodin 0.0106 g	Lowering blood Lipid	G20080005

#### 5.2.2 Snack foods

With the improvement of public health awareness, increased attention has been focused on the development of *L. lucidum*. To improve the utilization mechanism of *L. lucidum*, the food industry tries to combine *L. lucidum* polysaccharides or extracts with food ingredients to make various types of food rich in bioactive compounds, such as beverages, yogurt, fruit tea, wine, preserves, and fruits vinegar.

### 5.3 Animal feed additives

One major problem faced by the livestock and poultry industry is a loss of productivity because of ailments. Utilization of in-feed antibiotics is one solution to overcome infections and improve the health status of animals (Gao et al., 2017). Nevertheless, antibiotic residue in foodstuffs due to the administration of antibiotics in feed is considered an important reason for the rapid spread of antimicrobial resistance in humans. In light of this, seeking alternatives for in-feed antibiotics has become imperative. Among the possible alternatives to antibiotics, herbal medicine has attracted enormous interest in recent years given their beneficial effects on immune function, animal growth, and metabolism (Wang et al., 2021). There is a very long history of TCM which has been characterized as antimicrobials and antiseptics. Results from the current study have demonstrated that administration of *L. lucidum* aqueous extract in broilers significantly alleviated malondialdehyde concentration, elevated glutathione reductase activity, and enhanced antibody titers against Newcastle disease virus as well as spleen lymphocyte proliferation of broilers, indicating the potential value of *L. lucidum* as antibiotics’ substitute in the poultry industry (Ma et al., 2009). Furthermore, in laying hens, *L. lucidum* is shown to attenuate mortality, cracked-egg rate, and blood serum levels of triglycerides, cholesterol, low-density lipoprotein cholesterol, and alanine aminotransferase, enhance blood serum levels of high-density lipoprotein cholesterol, which suggested the tremendous potential of *L. lucidum* as the alternatives to antibiotics in poultry farming (Li et al., 2017). It was reported that the addition of *L. lucidum* into the diet at morning feed strongly improved blood antioxidant function, nutrient digestibility, and slaughtered body weight in sheep (Qiao et al., 2012).

### 5.4 Garden field

*Ligustrum lucidum* is a dramatic evergreen arbor tree, usually utilized as an ornamental tree for shade, and shelter, especially for hedging purposes, which can be conducted to a specific size and shape through regular pruning. This plant is regarded as an outstanding landscaping plant because it gives dense shade, grows rapidly even in poor soils, resists wind, pests, and air pollution and its flowers are attractive and heavily fragrant. Furthermore, *L. lucidum* is known for its health benefits, and it has been recognized as a potentially suitable plant for use as the general bioindicator of urban air pollution environments (Wei et al., 2018; Graziani et al., 2019).

### 5.5 Other application

Extensive eutrophication has led to the bloom of toxic cyanobacteria in major lakes in China. Microcystis aeruginosa (MA), as the most important kind of toxic cyanobacteria, is a vital threat to human and environmental health. *Ligustrum lucidum* has a long fruiting season which is from July to May of next year, and a large number of the fruits are unpicked almost every year. One study evaluated the effects of *L. lucidum* on the growth inhibition, cell integrity, and algicidal properties of MA. The result indicated that *L. lucidum* could be an excellent algicidal agent in the treatment of eutrophic water, ascribing to its strong inhibition on the growth of MA in the acute time. Consequently, utilization of the forgotten fruit may be an attractive, environmentally friendly, and cost-efficient option for cyanobacteria inhibition on eutrophic water (Varela et al., 2023; Zheng et al., 2024).

## 6 Safety

Although *L. lucidum* has been used as a critical tonic in different traditional medical systems worldwide for centuries. Until up to now, data on the systematic safety evaluation of this herb is still insufficient, toxicological experiments were seldom reported.

More recently, researchers have paid growing attention to respiratory allergies because they can cause illness and disability, as well as affect the quality of life (Vizuet-de-Rueda et al., 2022). Respiratory allergies influence humans in different parts of the world, leading to wide morbidity (Suanno et al., 2021). There is plenty of evidence that *L. lucidum*-derived pollen is one of the most prominent inhalant allergens contributing to respiratory allergic diseases worldwide given the ornamental plant introduced extensively (Mani et al., 2015). It is claimed that the pollen from *L. lucidum* causes wheezing, asthma, and eczema (Robledo-Retana, 2020). Additionally, in 2011, Dong et al. demonstrated that within 30 days after orally administering supercritical CO_2_ extract of *L. lucidum* fruit (at the dose of 0.75, 2.25, 3.75 g/kg) the behavior, hemogram, and blood component of the tested rats did not alter, nor caused any signs of genetic toxicity and subacute toxicity (Dong et al., 2011).

## 7 Conclusion and outlooks

Medicinal plants have long served as a natural treasure of bioactive components used to prevent and treat ailments. As an excellent medicinal plant with the homology of medicine and food, *L. lucidum* is rich in nutrients and active ingredients and, consequently, has a variety of biological properties such as anti-osteoporosis, antioxidant, liver protection, anti-inflammatory, and analgesic activity, as well as the effect on the central nervous system and glucose metabolism, especially excellent anti-tumor activity. The above researches partially shed light on the interacting underlying mechanism between *L. lucidum* and organisms and provides the theoretical and practical basis for part traditional applications. It is suggested that the following areas should be taken into consideration in future research.

With modern research tools applied, much in-depth study has been performed in *L. lucidum* for many years. Nonetheless, modern pharmacological research has been concentrated on the anti-osteoporotic and hepatoprotective activity with relatively weak applications in antioxidant and immunomodulatory activity. The molecular mechanisms of action, signaling pathways, and targets remain largely unknown. The active ingredients responsible for some activities of *L. lucidum* are far from clear, which strongly impeded the application of this herb. Therefore, the pharmacological mechanism of action of the bioactive components in *L. lucidum* should be studied in depth to provide a theoretical basis for their application in health products.

*Ligustrum lucidum* is typically applied in multi-herb formulation or integrated with conventional drugs in long-standing clinical applications, resulting in significant therapeutic benefits. However, most of the modern pharmacological and toxicological research has been generally performed on single *L. lucidum* or its main compositions, which critically ignored the effects induced by medicine interaction. According to the traditional applications, it is vital to explore the synergistic efficacy of this herb with drug use and enhance clinical efficacy and safety.

As a traditional medicine and food plant, toxicological studies and the detailed bioavailability, distribution, metabolism, and excretion of its main compositions in the body are still insufficient, thereby limiting its wider applications. Most toxicological research has focused on herbal medicine toxicity on healthy organisms, ignoring possible existing transformations in herb properties triggered by different pathological conditions. Therefore, further research on the toxicology and pharmacokinetics of *L. lucidum* should be strengthened. Nowadays, *L. lucidum* has limitations in the development and application of healthcare products. To ensure the long-term sustainable development prospects of the herb, further study should be closely linked to develop new products, and then continue to promote the development of *L. lucidum* in medicine, food, and health products, to achieve new values and connotations of *L. lucidum*.

## Figures and Tables

**Table:** 

**MFH**	Medicine food homology
**TCM**	Traditional Chinese medicine
**CFDA**	China Food and Drug Administration
**UPLC**	Ultra-performance liquid chromatography
**QTOFMS**	Quadrupole time-of-flight mass spectrometry
**EZP**	Erzhi Pill
**ZQFZ**	Zhenqi Fuzheng formula
**AD**	Alzheimer’s disease
**OPG**	Osteoprotegerin
**MAPK**	Mitogen-activated protein kinase
**AKT**	Protein kinase B
**ALT**	Alanine aminotransferase
**ALP**	Alkaline phosphatase
**AST**	Aspartate aminotransferase
**SCFAs**	Short-chain fatty acids
**CaSR**	Calcium-sensing receptor
**NF-КB**	Nuclear factor КB ligand
**ROS**	Reactive oxygen species
**BHT**	Butylated hydroxytoluene
**TC**	Total cholesterol
**SREBP-1c**	Sterol regulatory element binding protein-1c
**LXR-α**	Liver X receptor-α
**HCV**	Hepatitis C virus
**HCC**	Hepatocellular carcinoma
**NO**	Nitric oxide
**TNF-α**	Tumour necrosis factor-α
**OA**	Osteoarthritis
**IA**	Intra-articular
**mTOR**	Mammalian target of rapamycin
**Bcl-2**	B cell lymphoma 2
**Bax**	Bcl-2 associated X
**CytC**	Cytochrome-C
**MMP2**	Matrix metalloproteinase2
**PI3K**	Phosphoinositide 3-kinase
**PD**	Parkinson’s disease
**MPTP**	1-Methyl-4-phenyl-1, 2, 3, 6-tetrahydropyridine hydrochloride
**AChE**	Acetylcholinesterase
**LPS**	Lipopolysaccharide
**TLR4**	Toll-like receptor-4
**Collagen I**	Collagen type I
**BMMSCs**	Bone marrow mesenchymal stem cells
**NAFLD**	Nonalcoholic fatty liver disease
**TG**	Triglyceride
