# Supplementary material for: A review of botany, phytochemistry, pharmacology, and applications of the herb with the homology of medicine and food: Ligustrum lucidum W.T. Aiton
Source: Front Pharmacol. 2024 Jun 12;15:1330732. doi: 10.3389/fphar.2024.1330732 (PMC11199554; doi:10.3389/fphar.2024.1330732)
Supplement: Supplementary file 1 [file Table1.pdf]

Table S11 Chemical constituents isolated from *Ligustrum lucidum* W.T. Aiton

No.	Compound	Molecular formula	Appearance	Herb materials	Source	Reference
**Iridoid glycosides**						
1	Ligulucidumoside A	$C_{26}H_{34}O_{12}$	White powder	Fruits	75% ethanol extract	Zhang et al. (2013)
2	Ligulucidumoside B	$C_{19}H_{26}O_{13}$	White powder	Fruits	75% ethanol extract	Zhang et al. (2013)
3	Ligulucidumoside C	$C_{19}H_{26}O_{13}$	White powder	Fruits	75% ethanol extract	Zhang et al. (2013)
4	Nuezhenelenoliciside	$C_{31}H_{42}O_{17}$	Yellow solid	Fruits	70% ethanol extract	Qiu et al. (2018)
5	Isojaslanceoside B	$C_{26}H_{30}O_{14}$	Yellow solid	Fruits	70% ethanol extract	Qiu et al. (2018)
6	6′-O-trans-cinnamoyl-secologanoside	$C_{25}H_{28}O_{12}$	Yellow solid	Fruits	70% ethanol extract	Qiu et al. (2018)
7	Specnuezhenide	$C_{31}H_{42}O_{17}$	White Powder	Fruits	Ethanol extract	He et al. (2018)
8	Oleuropein	$C_{25}H_{32}O_{13}$	Brown powder	Fruits and leaves	Ethanol extract	Chen et al. (2020); Zhang et al. (2018)
9	Nuezhenoside G13	$C_{48}H_{64}O_{27}$	Light brown powder	Fruits	Ethanol extract	Chen et al. (2020); He et al. (2018)
10	Lucidumosides A	$C_{25}H_{34}O_{12}$	Colorless powder	Fruits	Ethanol extract	He et al. (2001)
11	Lucidumosides B	$C_{25}H_{34}O_{13}$	Colorless powder	Fruits	Ethanol extract	He et al. (2001)
12	Lucidumosides C	$C_{27}H_{36}O_{14}$	Amorphous powder	Fruits	Ethanol extract	He et al. (2001)
13	Lucidumosides D	$C_{27}H_{36}O_{13}$	Amorphous powder	Fruits	Ethanol extract	He et al. (2001)
14	Neonuezhenide	$C_{31}H_{42}O_{17}$	White crystalline powder	Fruits	Ethol extract	He et al. (2001)
15	Oleoside dimethyl ester	$C_{18}H_{26}O_{12}$	White powder	Fruits	Ethanol extract	He et al. (2001)

16	Isonuezhenide	$C_{32}H_{42}O_{17}$	White powder	Fruits	Ethol extract	He et al. (2001)
17	Nuezhenide	$C_{31}H_{42}O_{17}$	Amorphous powder	Fruits	95% ethanol extract	Fu et al. (2010)
18	Iso-oleonuezhenide	$C_{48}H_{64}O_{27}$	Colorless amorphous powder	Fruits	95% ethanol extract	Fu et al. (2010)
19	Methyloleoside 7-ethyl ester	$C_{19}H_{28}O_{11}$	Colorless amorphous powder	Fruits	95% ethanol extract	Fu et al. (2010)
20	G13	$C_{48}H_{64}O_{27}$	White powder	Fruits	95% ethanol extract	Fu et al. (2010)
21	Jaspolyside methyl ester	$C_{17}H_{24}O_{11}$		Fruits	95% ethanol extract	Fu et al. (2010)
22	Ligulucisides A	$C_{25}H_{28}O_{12}$	White amorphous powder	Fruits	70% ethanol extract	Pang et al. (2018)
23	Ligulucisides B	$C_{26}H_{30}O_{12}$	White amorphous powder	Fruits	70% ethanol extract	Pang et al. (2018)
24	Ligulucisides C	$C_{25}H_{30}O_{14}$	White amorphous powder	Fruits	70% ethanol extract	Pang et al. (2018)
25	Liguluciridoids A	$C_{13}H_{18}O_{6}$	Light yellow oil	Fruits	70% ethanol extract	Pang et al. (2018)
26	Liguluciridoids B	$C_{14}H_{22}O_{7}$	Light yellow oil	Fruits	70% ethanol extract	Pang et al. (2018)
27	2''-epifraxamoside	$C_{25}H_{30}O_{13}$	Colorless amorphous solid	Fruits	70% ethanol extract	Pang et al. (2018)
28	Excelsides B	$C_{31}H_{42}O_{17}$	White, amorphous powder	Fruits	70% ethanol extract	Pang et al. (2018)
29	Fraxamoside	$C_{25}H_{30}O_{13}$	Colourless amorphous powder	Fruits	70% ethanol extract	Pang et al. (2018)
30	Nuezhenidic acid	$C_{17}H_{24}O_{14}$	Off-white powder	Fruits	70% ethanol extract	Pang et al. (2018)
31	6'-*O*-*trans*-Cinnamoyl 8-epikingisidic acid	$C_{25}H_{28}O_{12}$	Amorphous powder	Fruits	Methanol extract	Pang et al. (2018)
32	Oleuropeinic acid	$C_{25}H_{30}O_{15}$	Colorless powder	Fruits	95% ethanol extract	Huang et al. (2011)
33	1'''-*O*-β-D-Glucosylformoside	$C_{31}H_{42}O_{17}$	Colorless powder	Fruits	95% ethanol extract	Huang et al. (2011)
34	Oleonuezhenide	$C_{48}H_{64}O_{27}$	White powder	Fruits	95% ethanol extract	Huang et al. (2011)
35	Ligusides A	$C_{48}H_{64}O_{27}$	Amorphous powder	Fruits	95% ethanol extract	Huang et al. (2011)

36	Ligusides B	$C_{48}H_{64}O_{27}$	Amorphous powder	Fruits	95% ethanol extract	Huang et al. (2011)
37	(8Z)-Nuezhenide A	$C_{31}H_{42}O_{17}$	Amorphous powder	Fruits	95% ethanol extract	Huang et al. (2011)
38	10-hydroxyligustroside	$C_{25}H_{32}O_{13}$		Leaves	Methanol extract	Kikuchi et al. (1999)
39	Ligustroside	$C_{25}H_{32}O_{12}$		Fruits and leaves	Methanol extract; Ethanol extract	Fu et al. (2010); Wang et al. (2017)
40	Ligustaloside A	$C_{26}H_{34}O_{13}$		Leaves	Methanol extract	Zhang et al. (2018)
41	Ligustaloside B	$C_{26}H_{34}O_{12}$		Leaves	Ethanol extract	Zhang et al. (2018)
42	8-demethyl-7-ketologanin	$C_{17}H_{24}O_{11}$	White amorphous powder	Leaves	Methanol extract	Kikuchi et al. (1999)
43	Iso-8-epikingiside	$C_{17}H_{34}O_{12}$	White amorphous powder	Leaves	Methanol extract	Kikuchi et al. (1999)
44	8-epikingiside	$C_{17}H_{24}O_{11}$		Leaves	Methanol extract	Kikuchi et al. (1999)
45	Kingiside	$C_{17}H_{24}O_{11}$		Leaves	Methanol extract	Kikuchi et al. (1999)
46	Isoligustrosidic acid	$C_{25}H_{30}O_{14}$	Amorphous powder	Fruits	Methanol extract	Aoki et al. (2012)
47	Oleopolynuzhenide A	$C65H_{86}O_{37}$	Amorphous powder	Fruits	Methanol extract	Aoki et al. (2012)
48	Nuzhenals A	$C_{10}H_{14}O_{5}$	Amorphous powder	Fruits	Methanol extract	Aoki et al. (2012)
49	Nuzhenals B	$C_{23}H_{30}O_{11}$	Amorphous powder	Fruits	Methanol extract	Aoki et al. (2012)
50	Nuzhenal C	$C_{11}H_{16}O_{5}$	White powder	Fruits	75% ethanol extract	Zhang et al. (2013)
51	Oleoside-7-ethyl-11-methyl ester	$C_{19}H_{28}O_{11}$	Colorless amorphous powder	Fruits	Aqueous extract	Chen et al. (2013)
52	10-hydroxyoleuropein	$C_{25}H_{32}O_{14}$	Light gray powder	Fruits	Ethanol extract	Yu et al. (2015)
53	6'-elenolylnicotiflorine	$C_{42}H_{54}O_{23}$	Colorless amorphous powder	Fruits	80% ethanol extract	Yu et al. (2015)
54	Nuezhengalaside	$C_{18}H_{28}O_{9}$	Colorless powder	Fruits	Aqueous extract	Chen et al. (2013)
55	Methyloleoside 7-ester	$C_{19}H_{28}O_{13}$	Colorless powder	Fruits	95% ethanol extract	Fu et al. (2010)

56	Oleoside-11-methyl ester	$C_{17}H_{24}O_{11}$	White crystalline powder	Fruits	80% ethanol extract	Yu et al. (2015)
57	6’”-acetylnicotiflorine	$C_{33}H_{44}O_{18}$	Colorless amorphous powder	Fruits	80% ethanol extract	Yu et al. (2015)
58	Nicotiflorine	$C_{31}H_{42}O_{17}$	Light yellow powder	Fruits	80% ethanol extract	Yu et al. (2015)
59	*p*-hydroxyphenethyl 7-β-D-glucosideelenolic acid ester	$C_{24}H_{32}O_{10}$	Colorless amorphous powder	Fruits	Ethanol extract	Liu et al. (2021)
60	4’,5’-(2’-hydroxy ligustrosidic acid) dimer	$C_{50}H_{56}O_{30}$	Amorphous powder	Fruits	Aqueous extract	Yu et al. (2015)
61	Oleonin	$C_{13}H_{18}O_{6}$	Yellow blocks	Fruits	70% ethanol extract	Huang et al. (2013)
62	Ibotalactone A	$C_{25}H_{28}O_{13}$	White powder	Leaves	Methanol extract	Kikuchi et al. (1999)
63	Ibotalactone B	$C_{25}H_{28}O_{14}$	White powder	Leaves	Methanol extract	Kikuchi et al. (1999)
**Terpenoids**						
64	3β-acetyl-20S,24R-dammarane-25-ene-24-hydroperoxy-20-ol	$C_{32}H_{54}O_{5}$	White powder	Fruits	80% ethanol extract	Xu et al. (2008)
65	20S,24R-dammarane-25-ene-24-hydroperoxy-3β,20-diol	$C_{30}H_{52}O_{4}$	White powder	Fruits	80% ethanol extract	Xu et al. (2008)
66	3β-acetyl-20S,25-epoxydammarane-24a-ol	$C_{32}H_{54}O_{4}$	White powder	Fruits	80% ethanol extract	Xu et al. (2008)
67	20S,25-epoxydammarane-3β,24a-diol	$C_{30}H_{52}O_{3}$	White powder	Fruits	80% ethanol extract	Xu et al. (2008)
68	20S-dammarane-23-ene-3β,20,25-triol	$C_{30}H_{52}O_{3}$	White powder	Fruits	80% ethanol extract	Xu et al. (2008)
69	Oleanolic acid	$C_{30}H_{48}O_{3}$	Light yellow powder	Fruits	Methanol extract	Xia et al., (2012)
70	Ursolic acid	$C_{30}H_{48}O_{3}$	Green powder	Fruits and leaves	Methanol extract	Wu et al. (2010); Xia et al. (2012)
71	3-*O*-cis-p-coumaroyl maslinic acid	$C_{39}H_{54}O_{6}$	White amorphous powder	Fruits	95% ethanol extract	Luo et al. (2020)
72	3-*O*-trans-p-coumaroyl maslinic acid	$C_{39}H_{54}O_{7}$	White amorphous powder	Fruits	95% ethanol extract	Luo et al. (2020)
73	Acetylursolic acid	$C_{32}H_{50}O_{4}$	White powder	Fruits	Methanol extract	Zhang et al. (2007)
74	Acetyl oleanolic acid	$C_{32}H_{50}O_{4}$	White powder	Fruits	Methanol extract	Xia et al. (2012)

75	β-Amyrin	$C_{30}H_{50}O$	White crystalline powder	Fruits and leaves	95% ethanol extract	Wu et al. (2010)
76	Ethyl oleanolate	$C_{32}H_{52}O_3$	White amorphous powder	Fruits	95% ethanol extract	Wu et al. (2010)
77	Methyl oleanolate	$C_{31}H_{50}O_3$	White amorphous powder	Fruits	70% ethanol extract	Wu et al. (2010)
78	2α-Hydroxyl oleanolic acid	$C_{30}H_{48}O_4$	White powder	Fruits	70% ethanol extract	Feng et al. (2011)
79	3β-*O*-cis-p-coumaroyl-2α-hydroxyloleanolic acid	$C_{39}H_{54}O_6$	White powder	Fruits	70% ethanol extract	Feng et al. (2011)
80	3β-*O*-trans-p-coumaroyl-2α-hydroxyloleanolic acid	$C_{39}H_{54}O_6$	White powder	Fruits	70% ethanol extract	Feng et al. (2011)
81	Ligustrin A	$C_{83}H_{146}O_{10}$		Fruits	70% ethanol extract	Machida et al. (1997)
82	α-Amyrin	$C_{30}H_{50}O$	White crystalline powder	Flowers	70% ethanol extract	Kikuchi et al. (1999)
83	Tormentic acid	$C_{30}H_{48}O_5$	White powder	Fruits	70% ethanol extract	Feng et al. (2011)
84	2α-Hydroxyl ursolic acid	$C_{30}H_{48}O_4$		Fruits	70% ethanol extract	Na et al. (2011)
85	Acetyl pomolic acid	$C_{32}H_{50}O_5$	White amorphous powder	Fruits	95% ethanol extract	Feng et al. (2019)
86	19α-Hydroxyl ursolic acid	$C_{30}H_{48}O_4$	White amorphous powder	Fruits	95% ethanol extract	Feng et al. (2019)
87	19α-Hydroxyl-3-acetyl- ursolic acid	$C_{32}H_{50}O_5$	White amorphous powder	Fruits	95% ethanol extract	Feng et al. (2019)
88	3α-*O*-cis-p-coumaroyl tormentic acid	$C_{39}H_{54}O_7$	White powder	Fruits	70% ethanol extract	Feng et al. (2011)
89	3β-O-trans-p-coumaroyl tormentic acid	$C_{39}H_{54}O_7$	White powder	Fruits	70% ethanol extract	Feng et al. (2011)
90	3-keto-oleanolic acid	$C_{30}H_{46}O_3$	White amorphous powder	Fruits	95% ethanol extract	Huang et al. (2011)
91	Lupeol	$C_{30}H_{50}O$	Colorless needle crystal	Leaves	Methanol extract	Wu et al. (2010)
92	Betulin	$C_{30}H_{50}O_2$	Colorless needle crystal	Leaves	Methanol extract	Wu et al. (2010)
93	Betulinic acid	$C_{30}H_{48}O_3$		Leaves	Methanol extract	Wu et al. (2010)
94	α-Ursolic acid methyl ester	$C_{31}H_{48}O_4$	White powder	Fruits	95% ethanol extract	Cheng et al. (2000)

95	Dammarendiol	$C_{30}H_{52}O_2$	Powder	Fruits	80% ethanol extract	Huang et al. (2011)
96	3-*O*-Acetyldammarenediol-II	$C_{32}H_{54}O_3$		Fruits	80% ethanol extract	Huang et al. (2011)
97	Dammarenediol II 3-*O*-palmitate	$C_{46}H_{82}O_3$	Colorless oily substance	Fruits	80% ethanol extract	Huang et al. (2011)
98	Dammar-24-ene-3β-acetoxy-20S-ol	$C_{32}H_{54}O_3$	White needle crystal	Fruits	80% ethanol extract	Huang et al. (2011)
99	Dammarenediol II	$C_{30}H_{52}O_2$	White Powder	Fruits	80% ethanol extract	Huang et al. (2011)
100	Dammar-25-ene-3β,20s,24s-triol	$C_{30}H_{52}O_3$	Powder	Fruits	80% ethanol extract	Huang et al. (2011)
101	Fouquierol	$C_{30}H_{52}O_3$	White amorphous powder	Fruits	95% ethanol extract	Huang et al. (2011)
102	Dammar-25-ene-3β,20,25-triol	$C_{30}H_{50}O_3$		Fruits	80% ethanol extract	Huang et al. (2011)
103	Oliganthas A	$C_{46}H_{82}O_5$	Colorless oily substance	Fruits	80% ethanol extract	Huang et al. (2011)
104	OcotillolII 3-*O*-palmitate	$C_{46}H_{82}O_4$	Colorless oily substance	Fruits	80% ethanol extract	Huang et al. (2011)
105	Cereotagaloperoxide	$C_{30}H_{52}O_4$	White solid	Fruits	70% ethanol extract	Huang et al. (2011)
**Phenylethanol compounds**						
106	Salidroside	$C_{14}H_{20}O_7$	Red brown powder	Fruits	Aqueous extract	Chen et al. (2013)
107	Verbascoside	$C_{29}H_{36}O_{16}$	Light yellow powder	Fruits	80% ethanol extract	Huang et al. (2011)
108	Echinacoside	$C_{35}H_{46}O_{20}$	White crystal	Fruits and leaves	Ethanol extract	Chen et al. (2020); Zhang et al. (2018)
109	Tyrosol	$C_8H_{10}O_2$	Gray white crystal	Fruits	Aqueous extract	Seo et al. (2017)
110	Hydroxytyrosol	$C_8H_{10}O_3$	Light yellow liquid	Fruits and leaves	Aqueous extract ; Ethanol extract	Chen et al. (2013); Zhang et al. (2018)
111	Tyrosyl acetate	$C_{10}H_{12}O_3$	Gray white solid	Fruits	Aqueous extract	Chen et al. (2013)
112	Osmanthuside H	$C_{14}H_{20}O_7$	Yellow powde	Fruits	80% methanol extract	Huang et al. (2011)
113	3,4-Dihydroxyphenylethanol-6'-caffeoyl-β-D-glucoside	$C_{23}H_{26}O_{11}$	White amorphous powder	Fruits	95% methanol extract	Huang et al. (2011)

114	3,4-Dihydroxyphenylethanol	$C_8H_{10}O_3$	Yellow liquid	Fruits	Methanol extract	Liu et al. (2021)
115	Isoverbascoside	$C_{29}H_{36}O_{16}$	White crystalline powder	Fruits	80% methanol extract	Li et al. (2017)
116	Cimidahurinine	$C_{14}H_{20}O_8$	White powder	Fruits	80% methanol extract	Li et al. (2017)
117	3,4-dihydroxyphenyl ethanol-O-β-D-glucoside	$C_{14}H_{20}O_8$	White amorphous powder	Fruits	95% methanol extract	Kikuchi and Yamauchi. (1999)
118	Acteoside	$C_{29}H_{36}O_{16}$	Colorless powder	Fruits	Ethanol extract	He et al. (2018)
119	Hydroxytyrosol glucoside	$C_{14}H_{20}O_8$		Leaves	Ethanol extract	Zhang et al. (2018)
**Flavonoids**						
120	Naringenin	$C_{15}H_{12}O_5$	Light yellow powder	Flowers	Acetone extract	Long et al. (2011)
121	Luteolin	$C_{15}H_{10}O_6$	Yellow power	Flowers and leaves	Acetone extract; Methanol extract	Long et al. (2011); Nerantzaki et al. (2011)
122	Apigenin	$C_{15}H_{10}O_5$	Yellow powder	Flower	Acetone extract	Long et al. (2011)
123	Quercetin	$C_{15}H_{10}O_7$	Yellow powder	Flower	Acetone extract	Long et al. (2011)
124	Rutin	$C_{27}H_{30}O_{16}$	Yellow powder	Leaves and flowers	Ethanol extract Acetone extract	Zhang et al. (2018); Long et al. (2011)
125	Apigenin-7-*O*-glucoside	$C_{21}H_{20}O_{10}$	Yellow powder	Leaves and flowers	Ethanol extract Acetone extract	Zhang et al. (2018); Long et al. (2011)
126	Apigenin-6ʹ-*O*-Acetyl-7-*O*-β-D-glucoside	$C_{23}H_{22}O_{11}$	Yellow powder	Fruits	Ethanol extract	Zhang et al. (2018)
127	Apigenin-7-*O*-rutinoside	$C_{27}H_{30}O_{14}$		Fruits	Ethanol extract	Zhang et al. (2018)
128	Kaemoterol	$C_{15}H_{10}O_6$	Yellow powder	Fruits	80% ethanol extract	Huang et al. (2011)
129	Apigenin 7-*O*-β-D-glucoside	$C_{21}H_{20}O_{10}$	Yellow granular crystal	Fruits	80% ethanol extract	Xu et al. (2008)
130	Luteolin-7-*O*-glucoside	$C_{21}H_{20}O_{11}$		Leaves	Ethanol extract	Zhang et al. (2018)

131	Luteolin-4`-*O*-glucoside	$C_{21}H_{20}O_{11}$		Leaves	Ethanol extract	Zhang et al. (2018)
132	Eriodictyol	$C_{15}H_{12}O_6$	White Powder	Fruits	Ethanol extract	Zhang et al. (2021)
133	Taxifoliol	$C_{15}H_{12}O_7$	Light yellow powder	Fruits	50% methanol	Zhang et al. (2021)
134	Ligustroflavone	$C_{33}H_{40}O_{18}$	Brown amorphous powder	Fruits	Ethanol extract	Kang et al. (2021)
**Volatile components**						
135	(+)-α-pinene	$C_{10}H_{16}$	Transparency liquid	Fruits	Aqueous extract	Xiao et al. (2019)
136	Camphene	$C_{10}H_{16}$	Colorless crystal	Fruits	Aqueous extract	Xiao et al. (2019)
137	Benzaldehyde	C_7H_6O	Transparency liquid	Fruits	Aqueous extract	Xiao et al. (2019)
138	4-ethenyl-pyridine	C_7H_7N	Red brown liquid	Fruits	Aqueous extract	Eberhardt et al. (2010)
139	(-)-β-pinene	$C_{10}H_{16}$	Colorless transparent liquid	Fruits	Aqueous extract	Xiao et al. (2019)
140	(+)-limonene	$C_{10}H_{16}$	Colourless liquid	Fruits	Aqueous extract	Xiao et al. (2019)
141	3-carene	$C_{10}H_{16}$	Colourless liquid	Fruits	Aqueous extract	Xiao et al. (2019)
142	2,5-dimethyl-benzenemethanol	$C_9H_{12}O$	Colourless liquid	Fruits	Aqueous extract	Xiao et al. (2019)
143	Nonanal	$C_9H_{18}O$	Brown liquid	Fruits	Aqueous extract	Xiao et al. (2019)
144	(-)-borneol	$C_{10}H_{18}O$	White powder	Fruits	Aqueous extract	Xiao et al. (2019)
145	4-terpineol	$C_{10}H_{18}O$	Light yellow liquid	Fruits	Aqueous extract	Xiao et al. (2019)
146	α-terpineol	$C_{10}H_{18}O$	Yellow crystals	Fruits	Aqueous extract	Xiao et al. (2019)
147	Citronelol	$C_{10}H_{20}O$	Light yellow liquid	Fruits	Aqueous extract	Xiao et al. (2019)
148	Cis-geraniol	$C_{10}H_{18}O$	Transparency liquid	Fruits	Aqueous extract	Xiao et al. (2019)
149	Geraniol	$C_{10}H_{18}O$	Light yellow liquid	Fruits	Aqueous extract	Xiao et al. (2019)

150	(E)-2-decena	$C_{10}H_{18}O$	Light yellow liquid	Fruits	Aqueous extract	Xiao et al. (2019)
151	Bornylacetate	$C_{12}H_{20}O_2$	White crystal	Fruits	Aqueous extract	Xiao et al. (2019)
152	(E, E)-2,4-decadienal	$C_{10}H_{16}O$	Yellowish Transparent Liquid	Fruits	Aqueous extract	Xiao et al. (2019)
153	γ-elemene	$C_{15}H_{24}$	Light yellow liquid	Fruits	Aqueous extract	Xiao et al. (2019)
154	β-copaene	$C_{15}H_{24}$		Fruits	Aqueous extract	Xiao et al. (2019)
155	Eugenol	$C_{10}H_{12}O_2$	Light yellow liquid	Fruits	Aqueous extract	Xiao et al. (2019)
156	Ylangene	$C_{15}H_{24}$	Colorless oil Liquid	Fruits	Aqueous extract	Xiao et al. (2019)
157	(-)-α-copaene	$C_{15}H_{24}$	Colorless clear liquid	Fruits	Aqueous extract	Xiao et al. (2019)
158	Dehydro-β-ionone	$C_{13}H_{18}O$	Pale Yellow Oil	Fruits	Aqueous extract	Xiao et al. (2019)
159	(-)-α-gurjunene	$C_{15}H_{24}$	Light yellow liquid	Fruits	Aqueous extract	Xiao et al. (2019)
160	β-cubebene	$C_{15}H_{24}$		Fruits	Aqueous extract	Xiao et al. (2019)
161	Caryophyllene	$C_{15}H_{24}$	Light yellow oily liquid	Fruits	Aqueous extract	Xiao et al. (2019)
162	Cis-β-copaene	$C_{15}H_{24}$		Fruits	Aqueous extract	Xiao et al. (2019)
163	Cyclodecadiene	$C_{15}H_{26}O$		Fruits	Aqueous extract	Xiao et al. (2019)
164	Cis-β-farnesene	$C_{15}H_{24}$		Fruits	Aqueous extract	Xiao et al. (2019)
165	γ-amorphene	$C_{15}H_{24}$		Fruits	Aqueous extract	Xiao et al. (2019)
166	Cadina-3,5-diene	$C_{15}H_{24}$		Fruits	Aqueous extract	Xiao et al. (2019)
167	Humulene	$C_{15}H_{24}$	Colourless liquid	Fruits	Aqueous extract	Xiao et al. (2019)
168	Epi-bicyclosesquiphellandrene	$C_{15}H_{24}$		Fruits	Aqueous extract	Xiao et al. (2019)
169	Calarene	$C_{15}H_{24}$		Fruits	Aqueous extract	Xiao et al. (2019)

170	γ-muurolene	$C_{15}H_{24}$		Fruits	Aqueous extract	Xiao et al. (2019)
171	1,5-cadinadiene	$C_{15}H_{24}$		Fruits	Aqueous extract	Xiao et al. (2019)
172	Germacrene D	$C_{15}H_{24}$		Fruits	Aqueous extract	Xiao et al. (2019)
173	α-muurolene	$C_{15}H_{24}$		Fruits	Aqueous extract	Xiao et al. (2019)
174	γ-cadinene	$C_{15}H_{24}$	Yellow oily liquid	Fruits	Aqueous extract	Xiao et al. (2019)
175	(+)-δ-cadinene	$C_{15}H_{24}$		Fruits	Aqueous extract	Xiao et al. (2019)
176	Epizonarene	$C_{15}H_{24}$		Fruits	Aqueous extract	Eberhardt et al. (2010)
177	Cubenene	$C_{15}H_{24}$		Fruits	Aqueous extract	Xiao et al. (2019)
178	α-cadinene	$C_{15}H_{24}$		Fruits	Aqueous extract	Xiao et al. (2019)
179	(E)-nerolidol	$C_{15}H_{26}O$		Fruits	Aqueous extract	Xiao et al. (2019)
180	γ-gurjunene	$C_{15}H_{24}$		Fruits	Aqueous extract	Xiao et al. (2019)
181	Caryophylleneoxide	$C_{15}H_{24}O$	White crystalline powder	Fruits	Aqueous extract	Xiao et al. (2019)
182	(-)-globulol	$C_{15}H_{26}O$	Colorless crystal	Fruits	Aqueous extract	Xiao et al. (2019)
183	(-)-spathulenol	$C_{15}H_{24}O$		Fruits	Aqueous extract	Xiao et al. (2019)
184	Epicubenol	$C_{15}H_{26}O$		Fruits	Aqueous extract	Eberhardt et al. (2010)
185	Junenol	$C_{15}H_{26}O$		Fruits	Aqueous extract	Eberhardt et al. (2010)
186	1,10-diepicubenol	$C_{15}H_{26}O$		Fruits	Aqueous extract	Xiao et al. (2019)
187	T-cadinol	$C_{15}H_{26}O$		Fruits	Aqueous extract	Xiao et al. (2019)
188	Torreyo	$C_{15}H_{26}O$		Fruits	Aqueous extract	Xiao et al. (2019)
189	α-cadinol	$C_{15}H_{26}O$		Fruits	Aqueous extract	Xiao et al. (2019)

190	(1R,7S,E)-7-isopropyl-4,10- dimethylenecyclodec-5-enol	$C_{15}H_{24}O$		Fruits	Aqueous extract	Xiao et al. (2019)
191	Farnesol	$C_{15}H_{26}O$	Colourless liquid	Fruits	Aqueous extract	Xiao et al. (2019)
192	Farnesal	$C_{15}H_{24}O$		Fruits	Aqueous extract	Xiao et al. (2019)
193	Methyl palmitate	$C_{17}H_{34}O_2$	Colorless solid	Fruits	Aqueous extract	Xiao et al. (2019)
**Others**						
194	*p*-coumaric acid	$C_9H_8O_3$	White powder	Leaves	Ethanol extract	Zhang et al. (2018)
195	β-sitosterol	$C_{29}H_{50}O$	White powder	Flowers	Acetone extract	Potenza et al., (2023)
196	Daucosterol	$C_{35}H_{60}O_6$	White powder	Flowers	Acetone extract	Potenza et al., (2023)
197	Gallic acid	$C_7H_6O_5$	Beige crystallization	Leaves	Methanol extract	Nerantzaki et al. (2011)
198	Caffeic acid	$C_9H_8O_4$	White powder	Leaves	Methanol extract	Nerantzaki et al. (2011)
199	Labiatenic acid	$C_{18}H_{16}O_8$	Red brown powder	Leaves	Methanol extract	Nerantzaki et al. (2011)
200	Procyanidin	$C_{30}H_{26}O_{13}$	Red brown powder	Fruits	Aqueous extract	Xiao et al. (2019)
201	Anthocyanidin	$C_{15}H_{11}ClO_6$	Purple powder	Flowers	Ethanol extract	Zhang et al. (2018)
202	Palmitic acid	$C_{16}H_{32}O_2$	White powder	Fruits	Aqueous extract	Eberhardt et al. (2010)
203	Stearic acid	$C_{18}H_{36}O_2$	White powder	Seeds	Methanol extract	Xie et al. (2009)
204	Oleic acid	$C_{18}H_{34}O_2$	Colorless liquid	Fruits	Aqueous extract	Eberhardt et al. (2010)
205	Linoleic acid	$C_{18}H_{32}O_2$	Light yellow liquid	Seeds	Methanol extract	Xie et al. (2009)
206	Linolenic acid	$C_{18}H_{30}O_2$	Yellow liquid	Seeds	Methanol extract	Xie et al. (2009)
